# CRISPR/Cas9 Screens Reveal that Hexokinase 2 Enhances Cancer Stemness and Tumorigenicity by Activating the ACSL4‐Fatty Acid *β*‐Oxidation Pathway

**DOI:** 10.1002/advs.202105126

**Published:** 2022-05-23

**Authors:** Hongquan Li, Junjiao Song, Yifei He, Yizhe Liu, Zhen Liu, Weili Sun, Weiguo Hu, Qun‐Ying Lei, Xin Hu, Zhiao Chen, Xianghuo He

**Affiliations:** ^1^ Fudan University Shanghai Cancer Center and Institutes of Biomedical Sciences Department of Oncology Shanghai Medical College Fudan University Shanghai 200032 China; ^2^ Key Laboratory of Breast Cancer in Shanghai Fudan University Shanghai Cancer Center Fudan University Shanghai 200032 China; ^3^ Collaborative Innovation Center for Cancer Personalized Medicine Nanjing Medical University Nanjing 211166 China

**Keywords:** ACSL4, cancer cell stemness, fatty acid *β*‐oxidation, hexokinase 2

## Abstract

Metabolic reprogramming is often observed in carcinogenesis, but little is known about the aberrant metabolic genes involved in the tumorigenicity and maintenance of stemness in cancer cells. Sixty‐seven oncogenic metabolism‐related genes in liver cancer by in vivo CRISPR/Cas9 screening are identified. Among them, acetyl‐CoA carboxylase 1 (ACC1), aldolase fructose‐bisphosphate A (ALDOA), fatty acid binding protein 5 (FABP5), and hexokinase 2 (HK2) are strongly associated with stem cell properties. HK2 further facilitates the maintenance and self‐renewal of liver cancer stem cells. Moreover, HK2 enhances the accumulation of acetyl‐CoA and epigenetically activates the transcription of acyl‐CoA synthetase long‐chain family member 4 (ACSL4), leading to an increase in fatty acid *β*‐oxidation activity. Blocking HK2 or ACSL4 effectively inhibits liver cancer growth, and GalNac‐siHK2 administration specifically targets the growth of orthotopic tumor xenografts. These results suggest a promising therapeutic strategy for the treatment of liver cancer.

## Graphical abstract

Hexokinase 2 (HK2) is a novel stimulus for liver cancer stem cells. The newly identified HK2‐acyl‐CoA synthetase long chain family member 4 (ACSL4)‐fatty acid β‐oxidation axis is an ideal therapeutic target for hepatocellular carcinoma (HCC), and GalNac‐conjugated siHK2 opens an avenue to develop a novel strategy of precision therapy for the treatment of HCC.

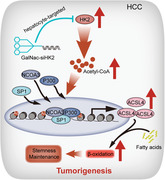

## 1 Introduction

Metabolic reprogramming is the major hallmark of tumorigenesis.^[^ ^1^ ^]^ Cancer cells often reprogram the metabolic pathways that control glycolysis, the tricarboxylic acid cycle (TCA), oxidative phosphorylation (OXPHOS), lipid synthesis, fatty acid *β*‐oxidation (FAO), glutaminolysis, and mitochondrial metabolism during carcinogenesis. The reprogramming of metabolism in cancer cells provides adequate materials, energy and redox balance for their proliferation and metastasis.^[^ ^2^ ^]^ Studies have shown that metabolic reprogramming is mediated by altered signal transduction directed by activated oncogenes and/or inactivated tumor suppressors.^[^ ^3^ ^]^ Interestingly, cancers arising in different tissues with diverse genetic alterations frequently have the same pattern of energy metabolism, which could provide potential strategies for targeted cancer therapy.^[^ ^4^ ^]^ However, the heterogeneity of most human tumors, which is caused by the activation of numerous oncogenes and/or the loss of multiple tumor suppressors, leads to a high complexity that results in the dependency of different tumors on distinct metabolic pathways.^[^ ^4^ ^]^ A comprehensive understanding of the altered metabolic genes in different human tumors is urgently needed.

Liver cancer is the fourth most common cause of cancer‐related death and ranks sixth in terms of incident cases worldwide.^[^ ^5^ ^]^ Hepatocellular carcinoma (HCC) is the most prevalent subtype of liver cancer. Common risk factors for HCC include hepatitis B virus (HBV) and hepatitis C virus (HCV) infections, alcohol intake, obesity, diabetes mellitus, nonalcoholic fatty liver disease (NAFLD), and nonalcoholic steatohepatitis (NASH).^[^ ^6^ ^]^ Even after surgical resection, the 5‐year survival rate of HCC patients remains poor due to high recurrence rates.^[^ ^7^ ^]^ System‐level approaches to analyze the genome‐wide transcriptome of 17 major cancer types revealed that HCC had the highest levels of transcriptional alterations.^[^ ^8^ ^]^ HCC onset and progression are frequently accompanied by rearrangements of metabolic pathways. Dysregulation of the hexosamine biosynthetic pathway, nucleotide metabolism, pentose phosphate pathway, and glycolysis has been reported in HCC based on transcriptomic and metabolomic analyses.^[^ ^9^ ^]^ Elucidation of the molecular functions and underlying mechanisms of the altered metabolic genes during liver carcinogenesis is important to fully reveal the pathogenesis of HCC and develop novel therapeutic strategies for its treatment.

Genome‐wide CRISPR/Cas9 screening is a powerful tool for identifying causal genes and studying the molecular mechanisms associated with specific phenotypes.^[^ ^10^ ^]^ Although in vitro studies are valuable for identifying the cell‐intrinsic properties of cancer cells, they cannot address problems involving complex interactions between multiple cell types.^[^ ^11^ ^]^ It is essential to investigate the resistance mediators and genotype‐specific fitness genes in vivo where cancers can develop in tissue microenvironments. In this study, we identified 67 metabolism‐related genes as oncogenic candidates for HCC using an in vivo CRISPR/Cas9 knockout screen that targeted 1121 differentially expressed genes. Cancer stem cells (CSCs) have been defined as a small subset of cancer cells within the tumor bulk that exhibit self‐renewal and differentiation,^[^ ^12^ ^]^ which contribute to tumor initiation, metastasis, relapse, and drug resistance.^[^ ^13^ ^]^ Recently, liver CSCs have been identified by several stem cell markers, including ALDH1A1, CD13, CD133, CD24, EpCAM, and CD90.^[^ ^14^ ^]^ Among the 67 oncogenic candidates, four metabolic enzymes (ACC1, ALDOA, FABP5, and HK2) were highly related to stem cell characteristics. HK2, a more efficient isoform for promoting aerobic glycolysis, plays a critical role in the maintenance and self‐renewal of liver CSCs through the acyl‐CoA synthetase long‐chain family member 4 (ACSL4)‐FAO pathway. Our findings indicate that HK2 can be an ideal therapeutic target and that GalNac‐siHK2 administration is a promising therapeutic strategy for specifically targeting HCC.

## 2 Results

### 2.1 In Vivo CRISPR/Cas9 Screens Identify Metabolic Genes That Determine HCC Cell Tumorigenicity

**Figure 1:**
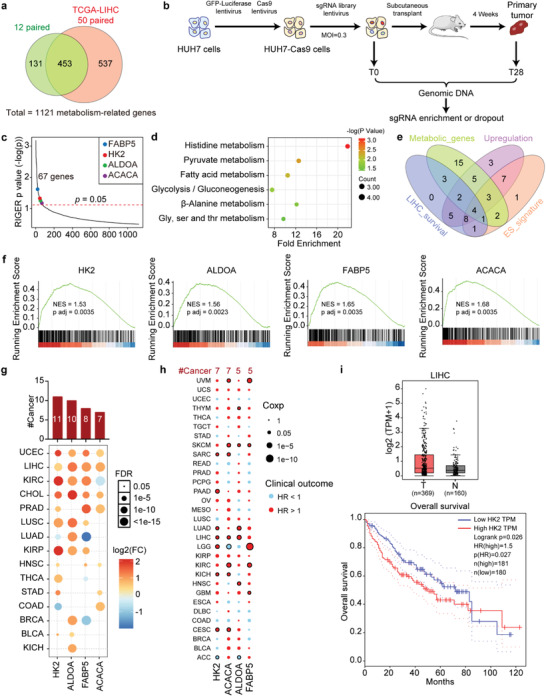
In vivo CRISPR/Cas9 screens identify metabolic genes that determine HCC cell tumorigenicity. a) Venn diagram showing the overlapping differentially expressed metabolism‐related genes between 12 paired HCC patients and 50 paired TCGA‐LIHC patients. b) A schematic diagram of the in vivo CRISPR/Cas9 screening. c) The genes were ranked on the basis of their corresponding RIGER *p* value for the metabolism‐related genes from the CRISPR/Cas9 screens. Analysis was performed using the RIGER algorithm. d) Enrichment of KEGG pathways. The horizontal axis shows the fold enrichment score in each signaling pathway. The color of the points in the figure represents the significance of the enrichment, and the size of the points represents the count of the genes enriched in each signaling pathway. e) Venn diagram showing the number of genes between the oncogenic metabolic genes identified from the CRISPR/Cas9 screens and the genes with upregulated expression, which were significantly associated with poor survival and an embryonic stem cell‐like gene expression signature in the TCGA‐LIHC cohort. Metabolic_genes: 35 of 67 metabolism‐related genes were metabolic enzyme genes, which directly participate in metabolic pathways. Upregulation: 37 of 67 metabolism‐related genes were upregulated in HCC. LIHC_survival: 24 of 67 metabolism‐related genes were associated with worse overall survival (OS) in HCC patients from TCGA‐LIHC. ES_signature: 27 of 67 metabolism‐related genes were highly associated with embryonic stem cell‐like gene expression signature. f) GSEA analysis of the previously defined ESC‐like module and metabolic gene expression in the TCGA‐LIHC cohort. The upper diagram shows the gene enrichment score as a dotted line. The horizontal axis shows each gene in this gene set, and the vertical axis shows the corresponding correlation coefficient with the indicated gene in patients from the TCGA‐LIHC cohort. Association between the enrichment of ES cell‐like gene expression signature and the expression of four metabolic genes in HCC tumor samples were shown by GSEA analysis. The mRNA level of the indicated metabolic gene was correlated significantly with that of the ES cell‐like gene. g) Upregulation patterns of four genes across different cancer types (*y* axis) compared with those of paired normal samples (FC > 1.5; t‐test corrected *p* < 0.05). The color intensity indicates the fold change, and the point size indicates the significance of the *p* value. Upper bars show the frequency of cancer types, with upregulation for each metabolic gene. h) Association of the expression of four genes with patient overall survival times based on univariate Cox proportional hazards models in different cancer types. Size denotes statistical significance at a given FDR. Color denotes the hazard ratio. Dots with black edges denote significant difference (Cox *p* < 0.05). i) HK2 expression between HCC patients and normal tissues from the TCGA LIHC dataset in the GEPIA database (top). Kaplan‐Meier curves show overall survival in the TCGA LIHC dataset in the GEPIA database (lower).

To comprehensively elucidate the functional roles of deregulated metabolic genes in HCC cell tumorigenicity, we selected 3224 metabolism‐related genes whose gene ontology (GO) annotations included metabolism‐related pathways. An integrative analysis was performed using transcriptomic data from the public TCGA‐LIHC database (including 50 paired HCC and noncancerous liver tissues) and our twelve paired HCC and noncancerous liver tissues. The results revealed that 1121 metabolism‐related genes were differentially expressed in HCC (**Figure** **1**a). Next, an in vivo CRISPR/Cas9 screening targeting the 1121 deregulated metabolism‐related genes was carried out (Figure 1b). A pooled sgRNA library, which contained 6726 sgRNAs targeting the 1121 metabolism‐related genes, 12 sgRNAs targeting two validated oncogenes, and 500 control sgRNAs (Table S1, Supporting Information), was constructed and transduced into HUH7 cells stably expressing Cas9‐GFP‐Luc at a multiplicity of infection (MOI) of 0.3. The transduced cells were selected with puromycin then propagated and subcutaneously transplanted into NOD/SCID mice. After we harvested genomic DNA from the primary tumors at 4 weeks after transplantation (Figure S1a, Supporting Information) with HUH7 cells, the sgRNA sequences were PCR‐amplified and measured by next‐generation sequencing to determine the enrichment in day 28 (T28) cells relative to day 0 (T0) cells (Figure 1b). After ranking the sgRNA representations in the groups (Table S1, Supporting Information), we identified 67 candidate oncogenic metabolism‐related genes that displayed significant sgRNA dropout by CRISPR screening (*p* value < 0.05) (Figure S1b–d, Supporting Information and Figure 1c). A KEGG pathway analysis showed that these 67 metabolism‐related genes were mainly enriched in the pyruvate, histidine, glycolysis/gluconeogenesis, Gly/Ser/Thr, fatty acid, and *β*‐alanine metabolic pathways (Figure 1d). Among these 67 oncogenic candidates, four metabolic enzyme genes, including FABP5, HK2, ALDOA, and ACACA, which show upregulated expression in HCC, were significantly associated with poor survival and embryonic stem (ES) cell‐like gene expression signatures^[^ ^15^ ^]^ (Figure 1e,f). Moreover, these four metabolic genes are usually increased in multiple types of cancer (Figure 1g), and their expression is linked to poor survival in cancer patients (Figure 1h). Notably, HK2 expression was increased in 11 cancer types and was associated with overall survival in seven cancers, including HCC^[^ ^16^ ^]^ (Figure 1g–i). This indicated that the dysregulation of HK2 is a common event in human cancer and may play a key role in cancer development and progression.

### 2.2 HK2 Is Highly Expressed in Liver CSCs and Is Essential for Maintaining Their Stemness and Self‐Renewal

**Figure 2:**
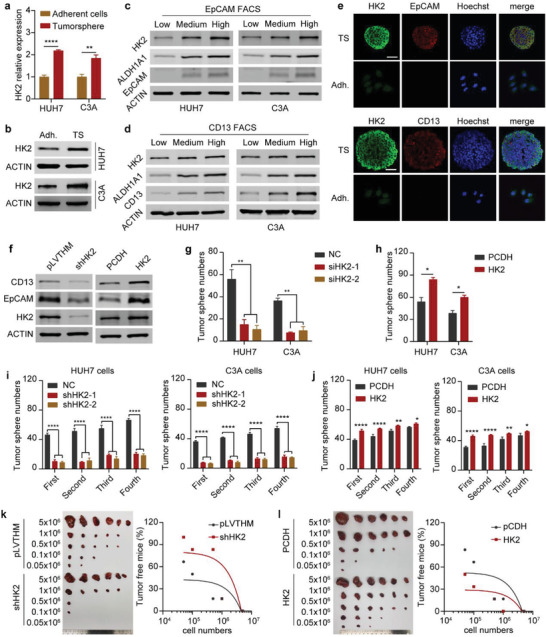
HK2 is highly expressed in liver CSCs and is essential for maintaining their stemness and self‐renewal. a) Real‐time qPCR showing HK2 mRNA expression (relative to ACTB) in adherent cells and tumorspheres of HUH7 and C3A cells. Data are represented as the mean ± SD (*n* = 3). Unpaired t‐test. b) Western blot measuring HK2 protein levels in adherent cells and tumorspheres of HUH7 and C3A cells. ACTIN was detected as a loading control. c) Western blot measuring HK2, ALDH1A1, and EpCAM protein levels in HUH7 or C3A cells after sorting by FACS with APC‐EpCAM antibody staining. ACTIN was detected as a loading control. d) Western blot measuring HK2, ALDH1A1, and CD13 protein levels in HUH7 and C3A cells after sorting by FACS with PE‐CD13 antibody staining. ACTIN was detected as a loading control. e) Immunofluorescence images of HK2 and EpCAM (up) and CD13 (down) levels in tumorsphere and adherent cells. All scale bars represent 50 µm. f) Western blot measuring CD13, EpCAM, and HK2 protein levels in HUH7 cells after HK2 knockdown or HK2 overexpression. Cells transfected with the empty vector (pLVTHM or PCDH) were used as a negative control. ACTIN was detected as a loading control. g) Tumorsphere formation assay of HUH7 and C3A cells after HK2 knockdown. Additionally, see the photographs in Figure S2i (Supporting Information). Data are represented as the mean ± SD (*n* = 2). One‐way ANOVA with multiple comparisons correction. h) Tumorsphere formation assay of HUH7 and C3A cells after HK2 overexpression. Additionally, see the photographs in Figure S2i (Supporting Information). Data are represented as the mean ± SD (*n* = 2). Unpaired t‐test. i) A serial tumorsphere formation assay of HUH7 (left) and C3A (right) cells after HK2 knockdown. Additionally, see the photographs in Figure S2j (Supporting Information). Data are represented as the mean ± SD (*n* = 2). One‐way ANOVA with multiple comparisons correction. j) A serial tumorsphere formation assay of HUH7 (left) and C3A (right) cells after HK2 overexpression. Additionally, see the photographs in Figure S2j (Supporting Information). Data are represented as the mean ± SD (*n* = 2). One‐way ANOVA with multiple comparisons correction. k) Limiting dilutions of HUH7 cells after HK2 knockdown were subcutaneously injected into BALB/c nude mice to observe tumor growth. Tumor sizes and tumor‐free mouse ratios are shown on the left and right, respectively. Additionally, see the statistical data‐sheet in Figure S2k (Supporting Information). Tumor‐free mouse ratios were analyzed with simple linear regression. l) Limiting dilutions of HUH7 cells after HK2 overexpression were subcutaneously injected into BALB/c nude mice to observe tumor growth. Tumor sizes and tumor‐free mouse ratios are shown on the left and right, respectively. Additionally, see the statistical data sheet in Figure S2l (Supporting Information). Tumor‐free mouse ratios were analyzed with simple linear regression. ns, non‐significant, *p < 0.05, **p < 0.01, ***p < 0.001, ****p < 0.0001.

Given that HK2 expression is often increased in HCC and is highly related to stem cell characteristics, we investigated whether HK2 could directly regulate the maintenance and self‐renewal of liver CSCs. Tumorspheres are ideal models that can satisfactorily enrich CSCs in vitro. The levels of the liver CSC markers ALDH1A1, CD13, CD24, EpCAM, Nanog, and OCT4 were significantly increased in the tumorspheres compared to those in the adherent HUH7 and C3A cells as shown by real‐time quantitative PCR (qPCR) (Figure S2a, Supporting Information), concomitantly the protein levels of ALDH1A1, CD13, EpCAM, Nanog, and OCT4 were much higher in the tumorspheres than in the adherent HUH7 and C3A cells (Figure S2b, Supporting Information), as shown in the western blot assays. This indicated that the tumorsphere formation assays showed enrichment for liver CSCs. In both the HUH7 and C3A cells, HK2 expression was increased in the tumorspheres at both the mRNA and protein levels compared with the adherent cells (**Figure** **2**a,b). Furthermore, fluorescence‐activated cell sorting (FACS) assays were used to sort the HUH7 and C3A cells using CSC markers, including EpCAM and CD13 (Figure S2c, Supporting Information). The FACS assays indicated that the CSC markers ALDH1A1 and HK2 showed increased expression in the high and medium EpCAM‐ and CD13‐expressing groups compared with the low expression groups in both HUH7 and C3A cells (Figure S2d,e, Supporting Information, Figure 2c,d). Moreover, immunofluorescence (IF) staining assays showed that the expression of the CSC markers EpCAM and CD13 as well as HK2 was much higher in the tumorspheres than in the adherent cells (Figure 2e). Taken together, these results indicate that HK2 is highly expressed in liver CSCs and is associated with CSC characteristics.

To further evaluate the role of HK2 in the maintenance of stemness and self‐renewal in liver CSCs, we designed two short hairpin RNAs (shRNAs) to knock down HK2 expression. We then cloned the full‐length CDS of HK2 into the PCDH vector to overexpress the HK2 protein in liver cancer cells (Figure S2f, Supporting Information). The mRNA and protein levels of CD13 and EpCAM were downregulated in HUH7 and C3A cells after HK2 knockdown, whereas they were upregulated after HK2 overexpression (Figure 2f and Figure S2g, Supporting Information). Moreover, FACS assays showed that the CD13‐ and EpCAM‐positive cells were decreased in HUH7 and C3A cells after HK2 knockdown (Figure S2h, Supporting Information). In contrast, the CD13‐ and EpCAM‐positive cells were increased in HUH7 and C3A cells after HK2 overexpression (Figure S2h, Supporting Information). Furthermore, the tumorsphere formation capability was dramatically impaired after HK2 knockdown in HCC cells (Figure 2g and Figure S2i, Supporting Information). In contrast, the overexpression of HK2 strongly enhanced the tumorsphere formation ability of HCC cells (Figure 2h and Figure S2i, Supporting Information). Serial tumorsphere formation assays proved that HK2 knockdown by shRNAs strongly decreased the tumorsphere formation capability (Figure 2i and Figure S2j, Supporting Information), whereas the high expression of HK2 significantly enhanced the tumorsphere formation capability of HCC cells (Figure 2j and Figure S2j, Supporting Information). Importantly, in vivo tumor propagation was substantially blocked after HK2 knockdown (Figure 2k and Figure S2k, Supporting Information), whereas it was strongly accelerated after HK2 overexpression (Figure 2l and Figure S2l, Supporting Information). Collectively, these data indicate that HK2 is essential for the self‐renewal and in vivo tumor propagation of liver CSCs.

### 2.3 ACSL4 Is Required for HK2‐Maintained Liver CSC Stemness

**Figure 3:**
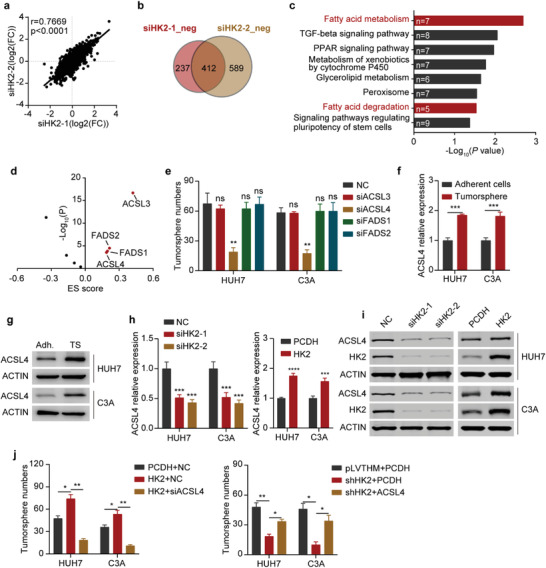
ACSL4 is required for HK2‐maintained liver CSC stemness. a) Correlation assay of RNA‐seq results of HUH7 cells after transfection with two independent HK2 siRNAs. b) Comparison of the genes with downregulated expression from the RNA‐seq results of HUH7 cells transfected with two independent HK2 siRNAs versus negative control (NC). A total of 412 genes showed downregulated expression in two independent HK2 siRNA‐transfected HUH7 cell lines. c) KEGG pathway analysis of the 412 genes on the DAVID website. d) Analysis of the embryonic stem cell‐like gene expression signature (ES score) of fatty acid metabolism‐ and fatty acid degradation‐related genes. Red dots show the positively correlated genes with a *p* value < 0.05 and score > 0. e) Tumorsphere formation assay of HUH7 and C3A cells after transfection with NC, ACSL3, ACSL4, FADS1, and FADS2 siRNAs. Additionally, see the photographs in Figure S3b (Supporting Information). Data are represented as the mean ± SD (*n* = 2). One‐way ANOVA with multiple comparisons correction. f) Real‐time qPCR showing ACSL4 mRNA expression (relative to ACTB) in adherent cells and tumorspheres of HUH7 and C3A cells. Data are represented as the mean ± SD (*n* = 3). Unpaired t‐test. g) Western blots measuring ACSL4 protein levels in adherent cells (Adh.) and tumorspheres (TS) of HUH7 and C3A cells. ACTIN was detected as a loading control. h) Real‐time qPCR showing ACSL4 mRNA expression (relative to ACTB) in HUH7 and C3A cells after HK2 knockdown or HK2 overexpression. Data are represented as the mean ± SD (*n* = 3). One‐way ANOVA with multiple comparisons correction. i) Western blots measuring ACSL4 and HK2 protein levels in HUH7 and C3A cells after HK2 knockdown and HK2 overexpression, respectively. ACTIN was detected as a loading control. j) Tumorsphere formation assay of HuH7 and C3A cells after HK2 overexpression and ACSL4 knockdown (left) and HK2 knockdown and ACSL4 overexpression (right). Data are represented as the mean ± SD (*n* = 2). One‐way ANOVA with multiple comparisons correction. ns, non‐significant, *p < 0.05, **p < 0.01, ***p < 0.001, ****p < 0.0001.

To determine the underlying molecular mechanism through which HK2 regulates liver CSC stemness, we performed RNA‐seq assays after silencing HK2 expression with two different siRNAs in HCC cells. The RNA‐seq results of the two siRNAs were highly consistent (**Figure** **3a** **)**, and the expression levels of 412 genes were commonly downregulated after HK2 knockdown (Figure 3b). Moreover, the KEGG pathway analysis of the 412 genes demonstrated that HK2 is strikingly involved in fatty acid metabolism and fatty acid degradation‐related gene regulation (Figure 3c). The results were further confirmed by real‐time qPCR assays (Figure S3a, Supporting Information). After evaluating the correlation of these fatty acid‐related genes with the ES cell‐like gene expression signatures in HCC, we found that four genes, including ACSL3, ACSL4, FADS1, and FADS2, were highly related to stem cell characteristics in liver cancer (Figure 3d). Furthermore, the knockdown of ACSL4, but not ACSL3, FADS1, or FADS2, could significantly impair the tumorsphere formation of HCC cells (Figure 3e and Figure S3b, Supporting Information), suggesting that ACSL4 may be involved in HK2‐regulated liver CSCs. The expression of ACSL4 was substantially higher in the tumorspheres than in adherent HCC cells at both the mRNA and protein levels, which was similar to the expression pattern of HK2 (Figure 3f,g). Furthermore, the expression of ACSL4 was reduced in the HK2‐silenced HCC cells, whereas its expression was elevated after HK2 overexpression (Figure 3h,i). This indicated that HK2 regulated ACSL4 expression in HCC cells. Moreover, the mRNA level of ACSL4 correlated significantly with those of HK2 in HCC patients (Figure S3c, Supporting Information). ACSL4 knockdown significantly decreased the tumorsphere formation in the HK2 overexpressing HCC cells, whereas the tumorsphere formation capability was recovered by ACSL4 overexpression in the HK2‐silenced HCC cells (Figure 3j and Figure S3d,e, Supporting Information). Taken together, these data indicate that ACSL4 is a downstream effector involved in HK2‐regulated CSC stemness in HCC.

### 2.4 ACSL4‐Activated FAO Mediates HK2‐Regulated Liver CSC Stemness

**Figure 4:**
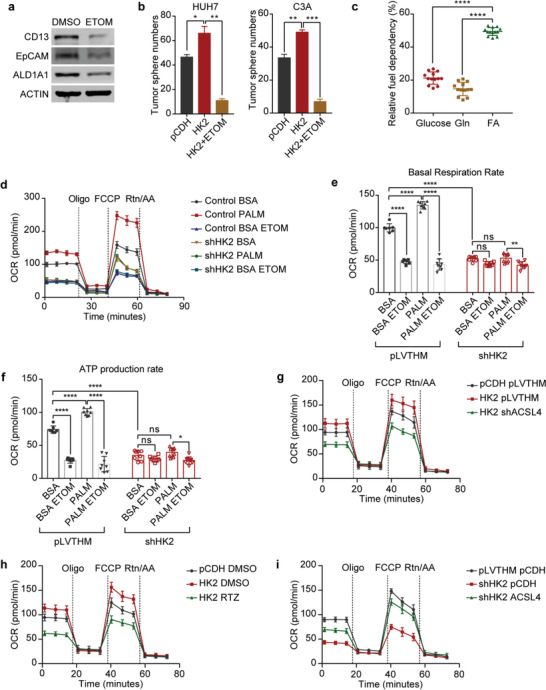
ACSL4‐activated FAO mediates HK2‐regulated liver CSC stemness. a) Western blots measuring CD13, EpCAM, and ALDH1A1 protein levels in HUH7 cells treated with DMSO or 10 × 10^−6^ m ETOM. ACTIN was detected as a loading control. b) Tumorsphere formation assay of HUH7 (left) and C3A (right) cells after HK2 overexpression and treatment with 10 × 10^−6^ m ETOM. Additionally, see the photographs in Figure S4c (Supporting Information). Data are represented as the mean ± SD (*n* = 2). One‐way ANOVA with multiple comparisons correction. c) Fuel dependency of HUH7 cells treated with BPTES (3 × 10^−6^ m), ETOM (4 × 10^−6^ m), and UK5099 (2 × 10^−6^ m). The graph shows the fraction of the OCR decrease induced by the inhibitors relative to the control. Box and whisker plots, *n* = 13 wells per condition, one‐way ANOVA with multiple comparisons correction. d) Time series of OCR measurements in HUH7 cells treated with pLVTHM BSA, pLVTHM PALM (palmitate), pLVTHM BSA ETOM, shHK2 BSA and shHK2 PALM, and shHK2 BSA ETOM by a Seahorse Metabolic Analyzer. Data are represented as the mean ± SD, *n* = 7–8 wells per condition. e) The (d) basal respiration rates of all groups. *n* = 7–8 wells per condition. One‐way ANOVA with multiple comparisons correction. f) The (d) mitochondrial ATP production rates (oligomycin‐sensitive respiration) of all groups. *n* = 7–8 wells per condition. One‐way ANOVA with multiple comparisons correction. g) Time series of OCR measurements in HUH7 cells after HK2 overexpression and ACSL4 knockdown by Seahorse Metabolic Analyzer. Data are represented as the mean ± SD, *n* = 6–8 wells per condition. h) Time series of OCR measurements in HUH7 cells after HK2 overexpression and RTZ treatment by a Seahorse Metabolic Analyzer. Data are represented as the mean ± SD, *n* = 6–8 wells per condition. i) Time series of OCR measurements in HUH7 cells after transfection with HK2 knockdown and ACSL4 overexpression by a Seahorse Metabolic Analyzer. Data are represented as the mean ± SD, *n* = 7–8 wells per condition. ns, non‐significant, *p < 0.05, **p < 0.01, ***p < 0.001, ****p < 0.0001.

ACSL4 is a fatty acid activation enzyme for FAO and lipid synthesis.^[^ ^17^ ^]^ FAO has been reported to regulate the maintenance of cancer cell stemness,^[^ ^18^ ^]^ which prompted us to hypothesize that ACSL4‐mediated FAO may be involved in HK2‐regulated liver CSC stemness. To test this hypothesis, we treated HCC cells with the pharmacological blocker etomoxir (ETOM), which is an inhibitor of carnitine palmitoyltransferase 1 (CPT‐1), to block the FAO process. The results proved that the expression of liver CSC markers, such as ALDH1A1, CD13, and EpCAM, was reduced at both the mRNA and protein levels after ETOM administration (Figure S4a, Supporting Information and **Figu** **re** **4a**). FACS assays further demonstrated that CD13‐positive cells were decreased after ETOM treatment (Figure S4b, Supporting Information). ETOM administration also significantly reduced the HK2‐promoted tumorsphere formation of HCC cells (Figure 4b and Figure S4c, Supporting Information).

Substrate dependency assays (Seahorse Mito Fuel Flex Test) based on oxygen consumption rate (OCR) responses to glutamine, glucose, and FA oxidation inhibitors (BPTES, UK5099, and ETOM, respectively) revealed that FAO was the preferred pathway for OXPHOS in HCC cells, which accounts for the largest part of oxidative respiration (Figure 4c and Figure S4d, Supporting Information). We can therefore use OCR to evaluate the FAO rate in HCC cells. Furthermore, OCR analyses demonstrated that the fatty acid substrate BSA‐conjugated palmitate (PALM) can significantly increase the basal respiration rates and mitochondrial ATP production, which can be blocked by the FAO inhibitor ETOM. This indicates that HCC cells can effectively utilize supplemented fatty acids (Figure 4d–f). In contrast, HK2‐defective HCC cells did not significantly change their respiration rates or mitochondrial ATP production. They also could not effectively oxidize fatty acids after PALM addition. These results revealed that HK2 knockdown led to FAO defects in HCC cells (Figure 4d–f). Next, we determined the role of ACSL4 in the HK2‐regulated FAO pathway. The overexpression of HK2 increased the OCR of HCC cells compared to the controls. When ACSL4 was knocked down or rosiglitazone (RTZ, ACLS4 inhibitor) was added, the OCR of the HK2‐overexpressing HCC cells was significantly decreased (Figure 4g,h and Figure S4e,f, Supporting Information). Additionally, a high expression of ACSL4 significantly rescued the OCR of the HK2 knockdown HCC cells (Figure 4i and Figure S4g, Supporting Information). As the expression of HK2 is important for fatty acid synthesis, we analyzed whether the source of fatty acids in FAO was due to glycolysis‐derived fatty acid synthesis or fatty acid uptake. We quantified fatty acids in HCC cells after HK2 or ACSL4 knockdown and found that the synthesis of many fatty acids was decreased in HK2‐silenced cells (Figure S4h, Supporting Information). This result suggests that HK2 is required for fatty acid synthesis. Intriguingly, many fatty acids accumulated in HCC cells after ACSL4 knockdown (Figure S4i, Supporting Information). Eleven fatty acids were decreased after HK2 knockdown but accumulated after ACSL4 knockdown in HCC cells. Twenty‐one fatty acids only accumulated after ACSL4 knockdown (Figure S4j, Supporting Information). These results suggested that fatty acids for FAO were from both synthesized fatty acids and fatty acid uptake. Since many fatty acids were extremely low in HCC cells (Figure S4k, Supporting Information), the palmitate acid (C16:0) may be a major fatty acid for FAO. Collectively, these data revealed that HK2 regulates liver CSC stemness through the ACSL4/FAO pathway.

### 2.5 Acetyl‐CoA Accumulation by HK2 Facilitates the Enhancer and Transcriptional Activity of ACSL4

**Figure 5:**
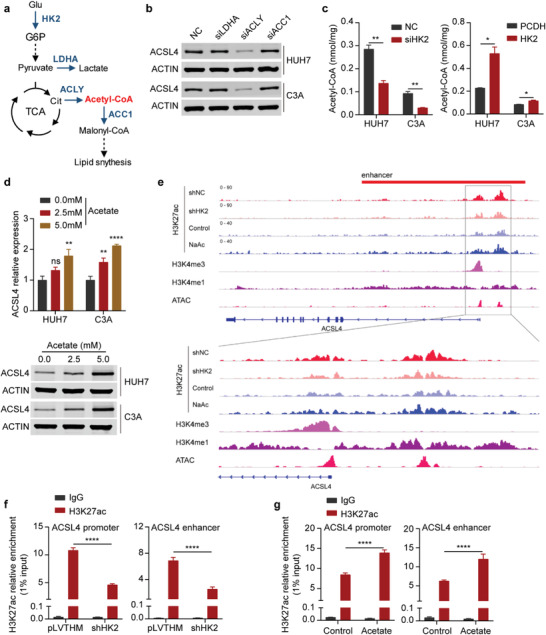
Acetyl‐CoA accumulation by HK2 facilitates the enhancer and transcriptional activity of ACSL4. a) Schematic diagram shows the relationship of glycolysis, the TCA cycle, and the fatty acid synthetic process. b) Western blots measuring ACSL4 protein levels in HUH7 (up) and C3A (down) cells transfected with NC, LDHA, ACLY, and ACC1 siRNAs. ACTIN was detected as a loading control. c) Measuring the amount of acetyl‐CoA in HUH7 and C3A cells after transfection with HK2 knockdown (left) or HK2 overexpression (right). Data are represented as the mean ± SD (*n* = 3). One‐way ANOVA with multiple comparisons correction. d) Upper: Real‐time qPCR showing ACSL4 mRNA expression (relative to ACTB) in HUH7 and C3A cells after treatment with acetate as indicated above. Data are represented as the mean ± SD (*n* = 3). One‐way ANOVA with multiple comparisons correction. Down: Western blot measuring ACSL4 protein levels in HUH7 and C3A cells after treatment with acetate as indicated above. ACTIN was detected as a loading control. e) ChIP‐seq assays showing the H3K27ac peak at the promoter and enhancer regions of ACSL4 in the negative control (shNC) and HK2 knockdown (shHK2) cells or control and acetate‐treated cells. The peaks of H3K4me3 and H3K4me1 represent the promoter and enhancer regions, respectively. f) ChIP‐PCR showing H3K27ac enrichment (relative to 1% input) at the ACSL4 promoter (left) and enhancer (right) regions in HUH7 cells after HK2 knockdown. Data are represented as the mean ± SD (*n* = 3). Two‐way ANOVA with multiple comparisons correction. g) ChIP‐PCR showing H3K27ac enrichment (relative to 1% input) at the ACSL4 promoter (left) and enhancer (right) regions in HUH7 cells after treatment with acetate (5 × 10^−3^ m). Data are represented as the mean ± SD (*n* = 3). Two‐way ANOVA with multiple comparisons correction. *p < 0.05, **p < 0.01, ***p < 0.001, ****p < 0.0001.

HK2 is the first factor in the glycolytic pathway, to which the TCA cycle and fatty acid synthesis are closely related (**Figure** 5a). Given that HK2 could upregulate ACSL4 expression and maintain liver CSC stemness by the ACSL4‐activated FAO pathway, we further explored the underlying mechanism by which HK2 facilitates ACSL4 transcription in HCC. We used siRNAs against LDHA, ACLY, and ACACA to assess whether ACSL4 expression depends on lactate, acetyl‐CoA, or fatty acid synthesis. The results revealed that siRNA against ACLY significantly decreased ACSL4 expression at both the mRNA and protein levels (Figure 5b and Figure S5a, Supporting Information). We further explored the effects of other glycolytic enzymes on ACSL4 expression by using siRNAs to target each enzyme (Figure S5b, Supporting Information). We found that the expression of ACSL4 was decreased after some of the glycolytic enzymes were silenced in HCC cells. This result suggested that other glycolytic enzymes were also involved in the regulation of ACSL4 expression and that the accumulation of acetyl‐CoA, which is produced by glycolysis, promoted the expression of ACSL4. Notably, HK2 knockdown decreased acetyl‐CoA levels, whereas HK2 overexpression increased acetyl‐CoA levels in HCC cells (Figure 5c). Our results confirmed that acetyl‐CoA could modulate the expression of ACSL4 in HCC cells. After treatment with acetate, which is the source of acetyl‐CoA, ACSL4 expression was increased in HCC cells at both the mRNA and protein levels (Figure 5d), indicating that HK2 regulates ACSL4 expression in a manner dependent on acetyl‐CoA levels in the cells. Next, we investigated the mechanism by which acetyl‐CoA regulates ACSL4 expression. Acetyl‐CoA is an acetyl group donor that modifies histone acetylation to control the activation of promoters and enhancers for gene expression.^[^ ^19^ ^]^ We conducted chromatin immunoprecipitation followed by sequencing (ChIP‐seq) and ChIP‐PCR to study the H3K27ac status of the ACSL4 promoters and enhancers regulated by HK2. The results showed that HK2 knockdown dramatically impaired the modification of H3K27ac (Figure 5e,f), whereas overexpression of HK2 increased the H3K27ac levels in both the promoter and enhancer regions of ACSL4 (Figure S5c, Supporting Information). Moreover, acetate administration increased the H3K27ac levels in the promoter and enhancer regions of ACSL4 (Figure 5g). Luciferase reporter assays showed that the enhancer region of ACSL4 had high transcriptional activity (Figure S5d, Supporting Information). Taken together, these data indicate that HK2 can increase the accumulation of acetyl‐CoA, thus leading to the H3K27ac modification of ACSL4 promoters and enhancers and increasing its expression.

### 2.6 EP300, NCOA3, and SP1 Are Required for the Activation of ACSL4 Transcription

**Figure 6:**
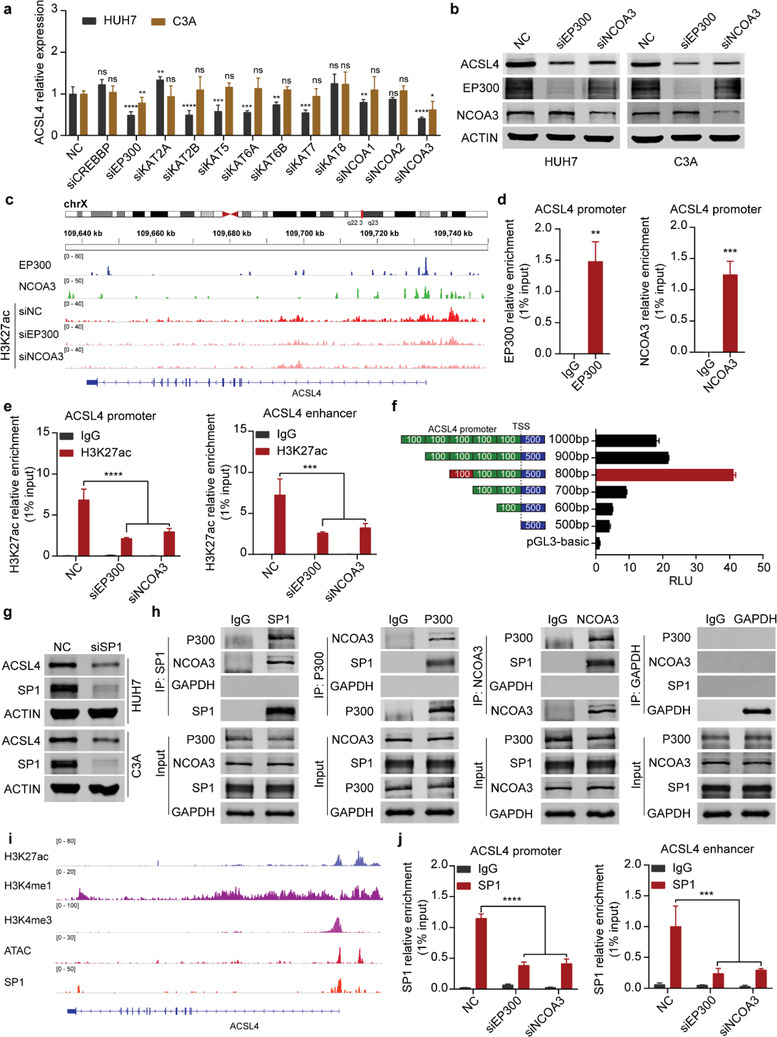
EP300, NCOA3, and SP1 are required for the activation of ACSL4 transcription. a) Real‐time qPCR showing ACSL4 mRNA expression (relative to ACTB) in HUH7 and C3A cells after transfection with a series of acetyltransferase siRNAs as indicated above. NC is the negative control. Data are represented as the mean ± SD (*n* = 3). One‐way ANOVA with multiple comparisons correction. b) Western blots measuring ACSL4 protein levels in HUH7 (left) and C3A (right) cells after transfection with EP300 or NCOA3 siRNAs. ACTIN was detected as a loading control. c) ChIP‐seq assays showing the peak enrichment of EP300 and NCOA3 at the ACSL4 promoter and enhancer regions in HUH7 cells. Peak enrichment of H3K27ac in the ACSL4 promoter and enhancer regions after EP300 or NCOA3 knockdown. d) ChIP‐PCR showing EP300 and NCOA3 enrichment (relative to 1% input) at ACSL4 promoter regions in HUH7 cells. e) ChIP‐PCR showing H3K27ac enrichment (relative to 1% input) at the ACSL4 promoter (left) and enhancer (right) regions in HUH7 cells after EP300 or NCOA3 knockdown. Data are represented as the mean ± SD (*n* = 3). Two‐way ANOVA with multiple comparisons correction. f) Luciferase assay measuring the active regions of the ACSL4 promoter in HUH7 cells by using a series of truncated regions of the ACSL4 promoter. Schematic diagram on the left. TSS, transcription start site. RLU, relative luciferase unit. Data are represented as the mean ± SD (*n* = 3). One‐way ANOVA with multiple comparisons correction. g) Western blots measuring ACSL4 protein levels in HUH7 (up) and C3A (down) cells after SP1 knockdown. ACTIN was detected as a loading control. h) Coimmunoprecipitation (Co‐IP) and western blots measuring SP1, P300, NCOA3, and GAPDH in HUH7 cells after Co‐IP using SP1, P300, NCOA3, and GAPDH antibodies. i) ChIP‐seq assays showing the peak enrichment of SP1 at promoter and enhancer regions of ACSL4. H3K27ac shows the promoter and enhancer regions of ACSL4. H3K4me3 and H3K4me1 show the promoter and enhancer regions of ACSL4, respectively. ATAC‐seq shows the open region of ACSL4 gene. j) ChIP‐PCR showing SP1 enrichment (relative to 1% input) at the ACSL4 promoter (left) and enhancer (right) regions in HUH7 cells after EP300 or NCOA3 knockdown. Data are represented as the mean ± SD (*n* = 3). Two‐way ANOVA with multiple comparisons correction. ns, non‐significant, *p < 0.05, **p < 0.01, ***p < 0.001, ****p < 0.0001.

To determine the acetyltransferase enzyme for the acetylation of H3K27 in the promoter and enhancer regions of ACSL4, we performed siRNA screening of major acetyltransferase enzymes in HCC cells. The results revealed that both EP300 and NCOA3 were required for ACSL4 transcription (Figure S6a, Supporting Information, **Figure** 6a,b). The expression level of ACSL4 was highly correlated with that of EP300 and NCOA3 in HCC (Figure S6b, Supporting Information). Furthermore, ChIP assays demonstrated that both EP300 and NCOA3 were enriched in the promoter and enhancer regions of ACSL4 (Figure 6c–e and Figure S6c, Supporting Information). Silencing EP300 and NCOA3 impaired the acetylation of H3K27 in both the promoter and enhancer regions of ACSL4 (Figure 6e). We further explored the possible transcription factors for ACSL4 transcription. Luciferase reporter assays of a series of ACSL4 promoter truncations demonstrated that the region ‐300–‐200 bp upstream from the transcription start site (TSS) was the core promoter region (Figure S6d, Supporting Information and Figure 6f). The possible transcription factors in the ‐300–‐200 bp promoter region were predicted, and their expression and correlation with ACSL4 in HCC were analyzed. The results indicated that four candidate transcription factors may be involved in ACSL4 transcriptional regulation (Figure S6e, Supporting Information). An siRNA screening assay showed that SP1 silencing significantly decreased ACSL4 expression at both the mRNA and protein levels (Figure S6f, Supporting Information and Figure 6g). A luciferase reporter assay also confirmed that SP1 silencing impaired ACSL4 promoter activity (Figure S6g, Supporting Information). We analyzed the interaction potential of SP1, EP300, and NCOA3 by using STRING (https://cn.string‐db.org). The results suggested that SP1, EP300, and NCOA3 could form a ternary complex (Figure S6h, Supporting Information). Intriguingly, the acetyltransferase enzymes EP300 and NCOA3 and transcription factor SP1 could form a ternary complex in HCC cells with GAPDH as a negative control (Figure 6h). ChIP assays showed that SP1 was enriched in the promoter and enhancer regions of ACSL4 (Figure 6i and Figure S6i, Supporting Information), and the enrichment was diminished after EP300 or NCOA3 silencing (Figure 6j). This suggests that EP300 and NCOA3 are required for SP1 recruitment to the promoter and enhancer regions of ACSL4 for its transcription.

### 2.7 GalNac‐siHK2 Administration Effectively Attenuates the Growth of Orthotopic Tumor Xenografts In Vivo

**Figure 7:**
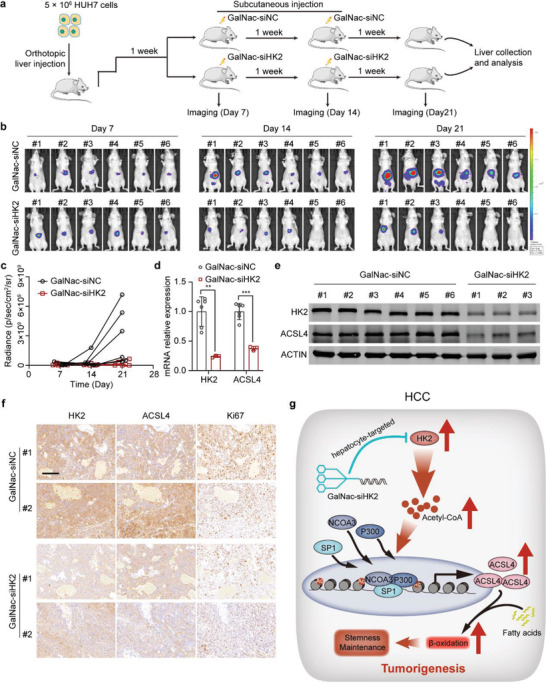
GalNac‐siHK2 administration effectively attenuates the growth of orthotopic tumor xenografts in vivo. a) A schematic diagram of the orthotopic tumor xenograft studies. HUH7 cells stably expressing GFP‐luciferase were injected into the livers of BALB/c nude mice in situ. After 7 d, mice were separated into two groups (*n* = 6) and treated with negative control (GalNac‐siNC) or GalNac‐siHK2 (5 mg kg^−1^). GalNac‐siHK2 was treated every 7 d. In vivo imaging to analyze the tumor growth. b) In vivo images showing the tumor growth of mice treated with the negative control and GalNac‐siHK2. c) Statistics of the radiance abundance of in vivo images. d) Real‐time qPCR showing HK2 and ACSL4 mRNA expression in tumor samples of the negative control and GalNac‐siHK2 treatment groups. Data are represented as the mean ± SD (*n* = 6 or 3, respectively). Unpaired t‐test. e) Western blots measuring HK2 and ACSL4 expression in tumor samples of the negative control and GalNac‐siHK2 treatment groups (*n* = 6 or 3, respectively). f) Immunohistochemistry was used to analyze the expression of HK2, ACSL4, and Ki67 in orthotopic tumor xenograft samples. Scale bar represents 100 µm. g) The working model shows that HK2 stimulates maintenance of stemness and self‐renewal of liver CSCs. Upregulated HK2 expression promoted the levels of acetyl‐CoA in HCC cells. HK2 accumulates acetyl‐CoA in HCC cells, induces promoter and enhancer histone acetylation, and activates the transcription of ACSL4, thus providing fatty acids for *β*‐oxidation. **p < 0.01, ***p < 0.001.

GalNac is a carbohydrate moiety that binds to asialoglycoprotein receptor 1 (ASGR1) with high affinity and facilitates the uptake of siRNAs into hepatocytes by endocytosis.^[^ ^20^ ^]^ GalNac‐conjugated siRNAs targeting hepatocyte‐deregulated genes have been approved by the FDA for the treatment of acute hepatic porphyria^[^ ^21^ ^]^ and have entered phase III trials.^[^ ^22^ ^]^ HK2 is highly expressed in HCC but barely expressed in healthy hepatocytes, which led us to hypothesize that GalNac‐siHK2 administration is a promising therapeutic strategy for targeting HCC. To test this hypothesis, we examined the expression of ASGR1 in multiple HCC cell lines. The results demonstrated that ASGR1 is highly expressed in the tumorspheres of HUH7 and C3A cells at both the mRNA and protein levels (Figure S7a–c, Supporting Information). Moreover, ASGR1 was highly expressed in HUH7‐formed orthotopic tumor xenografts and had similar levels in the normal liver tissues of mice. Metastatic tumors in mouse lungs also expressed high levels of ASGR1 (Figure S7d, Supporting Information), which suggested that HUH7‐formed orthotopic tumor xenografts are an ideal model for evaluating the therapeutic effect of GalNac‐siHK2. Metastatic tumors of HCC can also be targeted by GalNac‐siHK2 due to the expression of ASGR1 in HCC metastatic tumors. HUH7 cells stably expressing GFP‐luciferase were injected in situ into the livers of BALB/c nude mice. The mice were subcutaneously given the negative control (GalNac‐siNC) (*n* = 6) or GalNac‐siHK2 (5 mg kg^−1^) (*n* = 6). In vivo imaging analyses indicated that GalNac‐siHK2 treatment dramatically inhibited the xenograft growth of HUH7 cells (**Figure** 7a–c and Figure S7e, Supporting Information). As expected, the expression of HK2 and ACSL4 was dramatically decreased in the xenografts after GalNac‐siHK2 treatment at both the mRNA and protein levels, as determined by qPCR and western blot analyses (Figure 7d,e). Immunohistochemistry (IHC) of the xenografts also confirmed the results. The expressions of Ki67, which is the proliferative index of cancer cells, and the CSC markers were also dramatically reduced in the GalNac‐siHK2 treatment group compared with the GalNac‐siNC treatment group (Figure 7f and Figure S7f–h, Supporting Information). We further noticed that tumors were absent in 3 out of 6 mice on day 14. To investigate the mechanisms by which tumors became absent, we investigated the expression of the tumor necrosis marker, TNF*α*, and the apoptosis markers, cleaved Caspase‐3 and PARP, in the xenografts from the GalNac‐siNC and GalNac‐siHK2 groups. The results showed that TNF*α* was not expressed in the xenografts from two groups (Figure S7i, Supporting Information), and knockdown of HK2 did not regulate the expression of TNF*α* in liver cancer cells (Figure S7j,k, Supporting Information). Apoptosis was further activated in the GalNac‐siHK2 group, which indicated that the knockdown of HK2 may activate the apoptotic pathway. In conclusion, these data indicate that GalNac‐siHK2 administration is an attractive therapeutic strategy for targeting HCC with a high expression of HK2.

## 3 Discussion

Multiple metabolic genes are deregulated in human cancer, which reprograms the metabolic pathways responsible for sustained proliferation, invasion, metastasis, angiogenesis, and drug resistance.^[^ ^23^ ^]^ Liver cancer frequently shows altered metabolic genes in glucose metabolism, the pentose phosphate pathway, nucleotide metabolism, glutamine catabolism, fatty acid biosynthesis, and the bile acid pathway;^[^ ^24^ ^]^ however, the exact functional roles of many dysregulated metabolic genes in tumorigenicity are still unclear. In the present study, an in vivo CRISPR/Cas9 knockout screen targeting 1121 aberrant metabolism‐related genes showed that 67 oncogenic candidates directly contributed to the tumorigenicity of HCC and were mainly enriched in the pathways of glucose metabolism, fatty acid metabolism, and amino acid metabolism. Some of the genes have been reported to be involved in the development and progression of HCC, including HK2,^[^ ^25^ ^]^ fatty acid synthase (FASN),^[^ ^26^ ^]^ fatty acid‐binding protein 5 (FABP5),^[^ ^27^ ^]^ fructose‐bisphosphate aldolase A (ALDOA),^[^ ^28^ ^]^ and acetyl‐CoA carboxylase 1 (ACC1, encoded by *ACACA*).^[^ ^29^ ^]^ Moreover, some of the genes, such as guanylate kinase (GUK1), deoxyuridine 5'‐triphosphate nucleotidohydrolase (DUT), and carbonic anhydrase 5B (CA5B), were first reported to directly promote the tumorigenicity of HCC. Four metabolic enzymes, including ACC1, ALDOA, FABP5, and HK2, were strongly associated with stem cell characteristics. A small subset of cancer cells with stem cell properties exhibit self‐renewal properties and may account for cancer initiation, metastasis, therapy resistance, and recurrence.^[^ ^30^ ^]^ Our data showed that HK2 is a novel stimulus for liver CSCs and exerts a fundamental function in the maintenance of stemness and tumorigenicity in HCC.

HK2 belongs to the hexokinase family, which has four isoforms (HK1‐4).^[^ ^31^ ^]^ Hexokinase is the first rate‐limiting enzyme in aerobic glycolysis and can catalyze the conversion of glucose to glucose‐6‐phosphate (G‐6‐P). Under physiological conditions, HK2 is mainly expressed in adipocytes or muscle cells but is barely expressed in the liver. The dysregulation of HK2 has been observed in multiple types of cancer,^[^ ^32^ ^]^ including liver cancer.^[^ ^25^, ^33^ ^]^ HK2 was reported to interact with VDAC1 in the mitochondrial outer membrane and rewired metabolism to aerobic glycolysis.^[^ ^34^ ^]^ Under glucose starvation conditions, HK2 can contribute to autophagic induction by inhibiting mTORC1.^[^ ^32^ ^]^ In a model of KRAS‐driven lung cancer, HK2 decreased oxidative stress by increasing the activity of the PPP and stabilizing BACH1.^[^ ^35^ ^]^ Here, we showed that HK2 can increase the production of acetyl‐CoA and promote H3K27 acetylation of the promoter and enhancer regions of ACSL4 by the acetyltransferase enzymes EP300 and NCOA3. This recruited the transcription factor SP1 to induce ACSL4 expression in HCC. HK2‐induced ACSL4 led to FAO for the maintenance of stemness and self‐renewal in liver CSCs, which identified a previously uncharacterized molecular mechanism for HK2 in human cancer.

Fatty acid *β*‐oxidation (FAO) is a primary bioenergetic source. Cancer cells rely on FAO for proliferation, survival, stemness, drug resistance, and metastatic progression.^[^ ^36^ ^]^ It was reported that PPAR*δ* drives the expression of various FAO enzymes to promote FAO in hematopoietic stem cells.^[^ ^37^ ^]^ Another study reported that CD36‐positive leukemic stem cells displayed a higher FAO rate and more resistance to drugs than CD36‐negative counterparts, indicating that FAO activity was a determinant of cancer stem cell properties. Furthermore, the Leptin‐JAK/STAT3 pathway upregulated the expression of CPT1B, FAO activity, and stem cell self‐renewal in breast cancer.^[^ ^18b^ ^]^ In the context of HCC, the potential mechanisms by which FAO elevation maintains self‐renewal ability have been reported.^[^ ^38^ ^]^ Our results indicated that HK2 could also be contributed to the effect of FAO activity on CSC stemness maintenance.

HK inhibitors, such as 2‐deoxyglucose (2‐DG) and 3‐bromopyruvate (3‐BP), may be useful as therapeutic agents against HCC.^[^ ^39^ ^]^ Our results demonstrated that 2‐DG could inhibit the tumorsphere formation, cell proliferation, and orthotopic tumor xenograft growth of HCC cells (Figure S8, Supporting Information). ACSL4, which is the key downstream mediator of HK2, is presented as an attractive therapeutic target for HCC. This was because an inhibitor of ACSL4, RTZ, strongly suppressed the stemness and tumorigenicity of HCC cells (Figure S8e,f, Supporting Information). Moreover, 2‐DG and RTZ can function synergistically (Figure S8a–d, Supporting Information). These observations suggest that commonly prescribed drugs may serve as new therapeutic agents for the treatment of HCC. Intriguingly, RTZ, which is a thiazolidinedione (TZD), is an oral insulin‐sensitizing agent extensively used in the treatment of type 2 diabetes.^[^ ^40^ ^]^ The use of RTZ is associated with decreased liver cancer incidence in patients with diabetes.^[^ ^41^ ^]^ However, these inhibitors require high concentrations and can be toxic due to side and secondary effects. Furthermore, 2‐DG and 3‐BP do not have cell‐specific effects, which can target normal tissue and cause drug‐related liver toxicity.^[^ ^42^ ^]^ RTZ might be associated with an increase in the risk of myocardial infarction^[^ ^43^ ^]^ and may target ACSL4 in human arterial smooth muscle cells and macrophages.^[^ ^44^ ^]^ GalNac, a carbohydrate moiety, can specifically target hepatocytes by binding to the liver‐specific receptor ASGR1/2 with high affinity and can effectively mediate the uptake of siRNAs into hepatocytes. GalNac acts as a satisfactory siRNA delivery vehicle, and GalNac‐conjugated siRNAs against hepatocyte‐deregulated genes have been approved by the FDA for the treatment of various liver‐related diseases^[^ ^21^ ^]^ or have entered phase III clinical trials.^[^ ^22^ ^]^ HK2 is underexpressed in adult hepatocytes but is highly expressed in HCC. Targeting HK2 allows for the selective eradication of HCC with a reduced risk of side effects. We proposed that the HK2‐ACSL4 axis exists both in HCC CSCs and non‐CSCs. A lower expression of HK2 was associated with the lower expression of ACSL4 in non‐CSCs, while a higher expression of HK2 stimulated the HK2‐ACSL4‐FAO axis to maintain the stemness and self‐renewal abilities of CSCs. The present study used siRNA against HK2 conjugated to GalNac to target both HCC CSCs and non‐CSCs, and GalNac‐siHK2 effectively inhibited tumor xenograft growth in vivo. This offers an opportunity to specifically target HCC using HK2 overexpression.

Previously, DeWaal et al. found that HK2 ablation inhibited the proliferation, survival, and in vivo tumor growth of HCC cells. They also showed that HK2 deficiency markedly increased the susceptibility to cell death induced by sorafenib. These results support the role of HK2 as a tumor promoter in HCC progression. HK2 depletion was found to inhibit glycolysis and induce oxidative phosphorylation in HCC.^[^ ^25^ ^]^ In our study, we used an in vivo CRISPR/Cas9 knockout screen to identify HK2 as an oncogenic candidate for HCC, which was consistent with the results from previous studies. HK2 was significantly associated with embryonic stem (ES) cell‐like gene expression signatures in HCC, and we demonstrated that HK2 can facilitate the maintenance and self‐renewal of liver CSCs. HK2 enhances the accumulation of acetyl‐CoA and epigenetically activating the transcription of ACSL4, leading to an increase in FAO activity. DeWaal et al. focused on the effect of HK2 depletion on tumorigenesis and the metabolism of HCC cells, whereas we focused on the effect of HK2 on the maintenance and self‐renewal of liver CSCs. Our results, therefore, highlight a transcription‐dependent mechanism of HK2 in stemness regulation in liver cancer.

In conclusion, we identified 67 metabolism‐related genes as oncogenic candidates for HCC and determined that HK2 stimulates the maintenance of stemness and self‐renewal of liver CSCs. HK2 accumulates acetyl‐CoA in HCC cells, induces promoter and enhancer histone acetylation, and activates the transcription of ACSL4, thus providing fatty acids for *β*‐oxidation (Figure 7g). The newly identified HK2‐ACSL4‐FAO axis is an ideal therapeutic target for HCC, and GalNac‐conjugated siRNA against HK2 opens an avenue to develop a novel strategy for precision therapy in the treatment of HCC.

## 4 Experimental Section

### Characterization of the Expression Alterations and Survival Analyses

The mRNA expression profiles and clinical features of ≈10 000 patients across 33 human cancers were downloaded from TCGA data portal (http://gdac.broadinstitute.org/). Normalized gene expression data based on expectation maximization (RSEM). Student's t test was used to assess the differential expression between TCGA tumor and paired normal samples. Genes were considered differential expression between tumor and normal paired samples if the fold‐change >1.5 and t‐test FDR < 0.05. Only cancer types with ≥5 paired samples were included in these analyses. A Cox model and log‐rank test were used to assess whether metabolism‐related gene expression was associated with the OS times in cancer patients and considered FDR < 0.05 to indicate significance.

### Cell Culture

HEK293T/17 (ATCC, ATCC Number: CRL‐11268; RRID: CVCL_1926, 2019), HUH7 (ATCC, ATCC Number: RCB1366; RRID: CVCL_0336, 2019) and HepG2/C3A (C3A) (ATCC, ATCC Number: CRL‐10741; RRID: CVCL_1098, 2019) cells were grown at 37 °C and 5% CO_2_ in high glucose DMEM (Glucose, 4.5 g L^−1^) supplemented with 10% fetal bovine serum and antibiotics. For the tumorsphere formation assay, 3000 HUH7 and C3A cells or 5000 HUH7 and C3A cells were seeded in flat‐bottom ultralow attachment 6‐well plates (Corning, NY, USA) and cultured in DMEM/F12 (1:1) supplemented with B‐27 supplement (Thermo Fisher Scientific, IL, USA) and 20 ng mL^−1^ of EGF and FGF.

### CRISPR/Cas9 Screening

One performed by a customized library containing 7238 sgRNAs specifically targeting 1121 metabolism‐related genes (6 sgRNAs per gene), with 500 negative control sgRNAs and 12 positive control sgRNAs for validated oncogenes (Table S1, Supporting Information). The CRISPR/Cas9 knockout library was synthesized by Ranen (Shanghai, China).

HUH7 cells were transduced with the pooled sgRNA lentiviral library at a low MOI value of 0.3. To ensure both the efficiency and coverage of infection, a large‐scale spin‐infection of 1.5 × 10^8^ cells in 12‐well plates (Falcon, USA) was used, with 1.5 × 10^6^ cells per well. After 2 h of high‐speed centrifugation of each plate at 2000 rpm, the infection was complete, and the cells were moved into larger flasks (Falcon, USA). After 7 d of puromycin (Invitrogen, USA) selection, the surviving cells were considered to be the day 0 sample, and 3 × 10^7^ of these cells were stored for further processing. The remaining cells were counted, and 1 × 10^7^ cells per mouse were subcutaneously injected into NOD/Scidil2R*γ* ^−/−^ (NSG) mice (male, *n* = 5, age 5 weeks old, purchased from Charles River, Shanghai, China) for xenograft tumor formation. After 4 weeks, the xenograft tumor was collected and stored for further processing.

Genomic DNA of day 0 samples and xenograft tumor were extracted from each sample with the Qiagen Blood & Cell Culture Midi Kit (Qiagen, USA). sgRNA cassettes were PCR‐amplified from genomic DNA of each sample. The primer sequences used to amplify lentiCRISPR sgRNAs during the first PCR were as follows: F1, AATGGACTATCATATGCTTACCGTAACTTGAAAGTATTTCG; and R1, CTTTAGTTTGTATGTCTGTTGCTATTATGTCTACTATTCTTTCC. The primers used for the second PCR included an 8‐bp barcode for the multiplexing of different biological samples: F2, AATGATACGGCGACCACCGAGATCTACACTCTTTCCCTACACGACGCTCTTCCGATCT (index) tcttgtggaaaggacgaaacaccg; and R2, CAAGCAGAAGACGGCATACGAGATGTGACTGGAGTTCAGACGTGTGCTCTTCCGATCT (index) tctactattctttcccctgcactgt. The amplicons resulting from the second PCR were extracted with beads (Beckman Coulter, USA), quantified, mixed, and sequenced using a NextSeq 500 instrument (Illumina, USA).

The raw FASTQ files were demultiplexed using Geneious 7.0 (Biomatters Inc.) and processed such that they contained only the unique sgRNA sequence. The designed barcode sequences from the library were assembled into a mapping reference sequence to align the processed reads to the library. The reads were then aligned to the reference sequence using the “Map to Reference” function in Geneious 7.0. After alignment, the number of uniquely aligned reads for each library sequence was calculated. The number of reads of each unique sgRNA for a certain sample was normalized as follows: normalized read counts per unique barcode = reads per barcode/total reads for all barcodes in the sample × 10^6^ + 1. The sgRNA score was generated and ranked according to the depletion or enrichment of the normalized sgRNA counts.

### Classification of ES Score for Each Tumor Sample

An embryonic stem cell‐like gene expression signature was selected that has been shown to associate with embryonic stem (ES) cell identity in the expression profiles of various human tumor types.^[^ ^15^ ^]^ The ES score for each tumor sample was calculated by using gene set variation analysis^[^ ^45^ ^]^ based on 380 mRNA‐based ES signatures. Spearman's rank correlation was used to assess the correlation between ES scores based on different expression of genes and indicated genes.

### Gene Set Enrichment Analysis (GSEA)

To identify the “ES cell‐like gene expression signature”^[^ ^15^ ^]^ that are correlated with four metabolic genes expression in HCC tumor samples, GSEA was performed for HCC in TCGA dataset. In this analysis, GSEA was performed on the ranked protein‐coding gene (PCG) list based on the Spearmen's correlation coefficient with four metabolic genes expression using the clusterProfiler package in R (https://www.r‐project.org/).

### IF and IHC

For analysis of HK2, CD13, and EPCAM expression in the tumorsphere, tumorspheres were fixed in 4% paraformaldehyde. Tumorspheres were embedded in paraffin, and paraffin sections (3–4 µm thick) were used. Adherent cells were cultured in glass slices and fixed with 4% paraformaldehyde. The samples were blocked with 2% BSA (Sangon Biotech, Shanghai, China). The samples were incubated with HK2, CD13, or EPCAM antibody, which was diluted according to the manufacturer's instructions, at 4 °C overnight. This step was followed by incubation with secondary antibody conjugated with Alexa Fluor 488 or Alexa Fluor 555 (Beyotime, Shanghai, China). Hoechst 33342 (Beyotime, Shanghai, China) was used to stain the nuclei of the cells.

For analysis of HK2, ACSL4, Ki67, and ASGR1 expression in xenografts, paraffin sections (3–4 µm thick) were used. Slides were treated in 3% H_2_O_2_ for 15 min and blocked in 2% BSA in PBS for 30 min. The samples were incubated with HK2, ACSL4, Ki67, and ASGR1 antibodies, which were diluted according to the manufacturer's instructions, at 4 °C overnight. Secondary antibodies and DAB staining were used according to the DAB Horseradish Peroxidase Color Development Kit (Beyotime, Shanghai, China). Antibodies are shown in Table S2 (Supporting Information).

### RNA‐Seq and Analysis

Total RNA was extracted from cells using TRIzol reagent (Invitrogen, CA, USA) according to the manufacturer's protocol. For screening of the candidate RNAs, a VAHTS mRNA‐seq V3 Library Prep Kit (Vazyme, Nanjing, China) was used to build the RNA‐seq library. Transcript expression was analyzed using StringTie (version 1.2.3) and quantified by fragments per kilobase of exon per million fragments mapped (FPKM).

### ChIP Assay

Cultured cells were crosslinked using 1% formaldehyde. Crosslinking was terminated by adding glycine to a final concentration of 0.125 m. Cells were scraped off the dish, collected into a fresh 1.5 mL tube, and resuspended in ChIP lysis buffer supplemented with proteinase inhibitor (Bimake, Shanghai, China). Chromatin was sheared into 200–1000 bp fragments by sonication under the proper conditions. IgG or ChIP degree antibodies were added to Protein A/G magnetic beads (Bimake, Shanghai, China) and rotated at room temperature. After 30 min, the chromatin mixture was added to the beads, and the sample was rotated at 4 °C overnight. Then, the tube was subjected to a magnetic field to remove the supernatant, which contained nonspecific fragments. The beads were washed 4 times and then eluted using MinElute Spin Columns (Qiagen, Hilden, Germany). The primers used in the ChIP assay are listed in Table S2 (Supporting Information).

### ChIP‐Seq Analysis

For library construction, a DNA‐seq kit from NEB was used following the manufacturer's instructions. TBE PAGE‐gel size selection was performed for final library size selection to obtain ChIP‐seq libraries containing fragments of 250 to 500 bp. For ChIP‐seq analyses, 150‐bp paired‐end reads were aligned to the reference human genome using Bowtie with standard alignment parameters. PCR duplicates were marked with the Picard “Mark Duplicates” utility and removed from further analysis. Bam files were converted to BigWig files using deepTools. For ChIP‐seq analysis, peaks were identified using the MACS2 peak caller with the following parameters: ‐f BAMPE ‐keep‐dup all ‐g hs ‐q 0.01. Peaks were annotated with Homer. The peak distribution along genomic regions of genes of interest was visualized with IGV.

### Seahorse Metabolic Analyzer

For the Mito Fuel Flex Test, 15 000 cells per well of control and shHK2 HUH7 cells were seeded in a Seahorse XF96 cell culture plate and allowed to adhere overnight. The next day, OCR measurements were taken using a Seahorse XFe96 analyzer (Agilent, CA, USA) according to the manufacturer's protocol. One hour prior to measurement, the regular cell culture medium was replaced with assay medium made by supplementing the XF Base Medium with 1 × 10^−3^ m pyruvate, 2 × 10^−3^ m glutamine, and 10 × 10^−3^ m glucose. Cells were treated with BPTES (3 × 10^−6^ m), ETOM (4 × 10^−6^ m), or UK5099 (2 × 10^−6^ m). The following concentrations of each drug were used during OCR acquisition: oligomycin, 1.5 × 10^−6^ m; FCCP, 1 × 10^−6^ m; rotenone/antimycin A, 0.5 × 10^−6^ m.

For the fatty acid oxidation test, 15 000 control cells per well and shHK2 HUH7 cells were seeded in Seahorse XF96 cell culture plates and allowed to adhere overnight. The next day, OCR measurements were taken using a Seahorse XFe96 analyzer (Agilent, CA, USA) according to the manufacturer's protocol. One hour prior to measurement, the cell culture media was replaced with FAO assay medium, also called Krebs Henseleit Buffer (KHB) supplemented with 2.5 × 10^−3^ m glucose, 0.5 × 10^−3^ m carnitine, and 5 × 10^−3^ m HEPES at a final pH of 7.4. PALM was applied at a final concentration of 0.1 × 10^−3^ m just before the start of the assay. The following concentrations for each drug were used during OCR acquisition: ETOM, 10 × 10^−6^ m; oligomycin, 1.5 × 10^−6^ m; FCCP, 1 × 10^−6^ m; rotenone/antimycin A, 0.5 × 10^−6^ m.

For the Cell Mito Stress Test, 5000 cells per well of control and shHK2 HUH7 or control and HK2 overexpressing HUH7 cells were seeded in Seahorse XF96 cell culture plates and allowed to adhere overnight. The next day, the cells were transfected with HK2 overexpressing shACSL4 lentivirus or the ACSL4 inhibitor RTZ (10 × 10^−6^ m). Two days later, 1 h prior to measurement, the regular cell culture medium was replaced with assay medium made by supplementing the XF Base Medium with 1 × 10^−3^ m pyruvate, 2 × 10^−3^ m glutamine, and 10 × 10^−3^ m glucose. The following concentrations of each drug were used during OCR acquisition: oligomycin, 1.5 × 10^−6^ m; FCCP, 1 × 10^−6^ m; rotenone/antimycin A, 0.5 × 10^−6^ m.

### GalNac‐siRNA Synthesis

The siRNA sequence of HK2 is CTGGCTAACTTCATGGATA. The GalNac ligand was introduced at the 3' end of the sense strand of the siRNA using a 3'‐GalNac CPG analog. The GalNac‐siRNA was synthesized and purchased from Huzhou Hippo Biotechnology Co., Ltd., Zhejiang, China.

### Animal Studies—Diluted Xenograft Tumor Formation

For the tumor‐initiating capacity assay in vivo, 0.05 × 10^6^, 0.1 × 10^6^, 0.5 × 10^6^, 1 × 10^6^, and 5 × 10^6^ cells were injected into BALB/c nude mice. Four weeks later, tumor formation was quantified, followed by calculation of the ratios of tumor‐free mice and tumor‐initiating cells.

### Animal Studies—Orthotopic Tumor Xenograft and In Vivo Imaging Studies

5 × 10^6^ HUH7 cells, which stably express GFP‐luciferase, were injected into each mouse to establish an orthotopic liver tumor xenograft. The mice were imaged every week with an IVIS Lumina LT Series III in vivo imaging system (PerkinElmer, Waltham, MA, USA) under anesthesia by isoflurane (RWD, Shenzhen, China) inhalation after intraperitoneal injection of 150 mg kg^−1^ D‐luciferin (Yeasen, Shanghai, China). Bioluminescence data were analyzed using Living Image software (PerkinElmer, Waltham, MA, USA). After one week of tumor establishment, bioluminescence intensity was used to randomize mice into two groups to ensure similar bioluminescence levels and received a subcutaneous injection of GalNac‐NC or GalNac‐siHK2 (5 mg kg^−1^).

All animal experiments were performed in accordance with protocols approved by the Institutional Animal Care and Use Committee of Fudan University (permission number: FUSCC‐IACUC‐S20210139), Shanghai, China.

### Statistical Analysis

Each experiment was performed with at least three independent replicates, and the results are expressed as the mean ± SD. Student's t‐tests (two‐tailed) and one‐way analysis of variance (ANOVA) were used to compare the means of two or more samples unless otherwise indicated. A *P*‐value < 0.05 was considered significant. All statistical analyses were performed using the GraphPad Prism 8 (GraphPad Software, CA, USA). The details of statistical analyses are presented in the figure legends.

## Conflict of Interest

The authors declare no conflict of interest.

## Author Contributions

H.L. and J.S. contributed equally to this work. X.H., H.L., and Z.C. conceived and designed the study. H.L., J.S., W.S., and X.H. developed and performed the experiments. H.L., J.S., Y.L., Y.H., Z.L., and W.S. acquired the data. H.L., J.S., and Z.C. analyzed the data. H.L., Z.C., W.H., Q.‐Y.L., and X.H. wrote and revised the manuscript. All authors read and approved the final manuscript.

## Supporting information

Supporting Information

Supplemental Table 1

Supplemental Table 2

## Data Availability

The sequencing data that support the findings of this study are listed in NCBI Gene Expression Omnibus (Accession no: GSE184798, GSE184797, and GSE184762). Other data that support the findings of this study are available from the corresponding author upon reasonable request.
