# Supplementary material for: CRISPR/Cas9 Screens Reveal that Hexokinase 2 Enhances Cancer Stemness and Tumorigenicity by Activating the ACSL4‐Fatty Acid β‐Oxidation Pathway
Source: Adv Sci (Weinh). 2022 May 23;9(21):2105126. doi: 10.1002/advs.202105126 (PMC9313492; doi:10.1002/advs.202105126)
Supplement: Supplementary file 1 — Supporting Information [file ADVS-9-2105126-s003.pdf]

ADVANCED SCIENCE
Open Access

Supporting Information

for *Adv. Sci.*, DOI 10.1002/advs.202105126

CRISPR/Cas9 Screens Reveal that Hexokinase 2 Enhances Cancer Stemness and Tumorigenicity by Activating the ACSL4-Fatty Acid β-Oxidation Pathway

Hongquan Li, Junjiao Song, Yifei He, Yizhe Liu, Zhen Liu, Weili Sun, Weiguo Hu, Qun-Ying Lei, Xin Hu, Zhiao Chen and Xianghuo He**

Supplemental Information

CRISPR/Cas9 screens reveal that hexokinase 2 enhances cancer stemness and tumorigenicity by activating the ACSL4-fatty acid β-oxidation pathway

Hongquan Li[1], Junjiao Song[1], Yifei He[1], Yizhe Liu[1], Zhen Liu[1], Weili Sun[2], Weiguo Hu[1, 2], Qun-Ying Lei[1], Xin Hu[2], Zhiao Chen[1, *], Xianghuo He[1, 2,3, *]

[1]Fudan University Shanghai Cancer Center and Institutes of Biomedical Sciences; Department of Oncology, Shanghai Medical College, Fudan University, Shanghai 200032, China.

[2]Key Laboratory of Breast Cancer in Shanghai, Fudan University Shanghai Cancer Center, Fudan University, Shanghai 200032, China.

[3]Collaborative Innovation Center for Cancer Personalized Medicine, Nanjing Medical University, Nanjing 211166, China.

These authors contributed equally to this work: Hongquan Li, Junjiao Song.

[#]Corresponding Authors:

Xianghuo He, E-mail: xhhe@fudan.edu.cn or Zhiao Chen, E-mail: zachen@fudan.edu.cn, Fudan University Shanghai Cancer Center and Institutes of Biomedical Sciences; Shanghai Medical College, Fudan University, 302 Rm., 7# Bldg., 270 Dong An Road, Shanghai 200032, China. Tel: 86-21-34777329; Fax:

86-21-64172585.

Supplemental Materials and Methods

siRNA Transfection

All siRNAs or negative control (NC) were synthesized by RiboBio (Guangzhou, China). The sequences of all siRNAs are shown in **Supplemental Table S2**. Before siRNA transfection, around 0.4 million HUH7 cells or 0.6 million C3A cells were seeded in to 6 well plate per well. Next day, cells in each well were transfected with 9μl of siRNA or NC (10μM) using Lipofectamine RNAiMAX (Invitrogen, CA, USA) according to the manufacturer's instructions. For some siRNAs of genes, mix the three independent siRNA (3μl each) for one target gene. After 24 hours transfection, the cells were harvested for further experiments. For RNA-seq or ChIP-PCR, total RNA was extracted after 48 hours transfection.

Plasmid Construction, Lentiviral Production and Stable Cell Line Set Up

To downregulate HK2 or ACSL4 expression in liver cancer cells, shRNAs of HK2 or ACSL4 were synthesized and cloned into PLVTHM lentiviral vector. The shRNA oligos are showed in **Supplemental Table S2**. The empty PLVTHM vectors were used as control. To overexpress HK2 or ACSL4 in liver cancer cells, we cloned the ORF of HK2 or ACSL4 into the PCDH-Puro lentiviral vector. The clone primers are shown in **Supplemental Table S2**.

To generate lentiviruses, the lentiviral vector, packaging plasmid (psPAX2), and

VSV-G envelope plasmid (pMD2.G) were cotransfected into HEK293T/17 cells using Lipofectamine 2000 (Invitrogen, CA, USA). The psPAX2 and pMD2.G plasmids were gifts from Dr. Didier Trono (Ecole Polytechnique Fédérale de Lausanne, 1015 Lausanne, Switzerland). Renew the medium after 8 hours later. Supernatants were collected after another 48 hours and store at -80°C before use.

To set up HK2 or ACSL4 knocking down stable cell lines, HUH7 or C3A cells were transfected with shHK2 or shACSL4 lentivirus, renew the medium after 8 hours later, for another 24 hours, sorting the positive cells which stable expressing GFP with FACS. To set up HK2 or ACSL4 overexpressed stable cell line, HUH7 or C3A cells were transfected with HK2 or ACSL4 overexpression lentivirus, renew the medium after 8 hours later, for another 24 hours, screening the positive cells with 1μg/ml of puromycin.

RNA Isolation, Reverse Transcription PCR (RT-PCR) and Real Time Quantitative PCR (qPCR)

Total RNA was extracted using Trizol reagent (Invitrogen, CA, USA) according to the manufacturer’s protocol. cDNA was synthesized by PrimeScript RT Reagent Kit (TaKaRa, Tokyo, Japan) according to the manufacturer’s protocol. SYBR Premix (TaKaRa, Tokyo, Japan) was used to detect the expression levels of genes of interest. Real time quantitative PCR (qPCR) primers used in this study are shown in **Supplemental Table S2**.

Luciferase Assay

The promoter or enhancer regions of ACSL4 were amplified from HUH7 cell genomic DNA and was cloned into the pGL3-basic or pGL3-Promoter vector respectively. The clone primers were shown in **Supplemental Table S2**. For analysis of promoter or enhancer activities, HUH7 cells were seeded into 96-well plates at a density of 5000 cells per well. After 24 h, 5 ng of pRL-TK, 100 ng of pGL3-basic or pGL3-ACSL4-promoter, and 5 pmol of NC or SP1 siRNA were transfected into each well of a 96-well plate; 100 ng pGL3-Promoter or pGL3-ACSL4-enhancer were transfected into each well of a 96-well plate. Firefly and Renilla luciferase activities were measured using the dual-luciferase reporter assay system (Promega, WI, USA).

Western Blotting Analysis

Proteins were separated by SDS-PAGE and transferred to nitrocellulose membranes (GE, CT, USA). The membranes were blocked with 5% nonfat milk and then incubated with primary antibodies overnight at 4°C. Antibodies used in this study are shown in **Supplemental Table S2**. Membranes were incubated with DyLight 800- or DyLight 680-conjugated secondary antibodies for 1 hour at room temperature. Membranes were scanning by Odyssey CLx Imaging System (LI-COR, Nebraska, USA).

Tumorsphere Formation Assay

For tumorsphere formation assay, 3,000 cells or 5,000 cells of HUH7 and C3A respectively were seeded in Flat Bottom Ultra-Low Attachment 6 well plate (Corning, NY, USA) and cultured in DMEM/F12 (1:1) supplemented with B-27 Supplement

(Thermo Fisher Scientific, IL, USA), 20 ng/ml of EGF and FGF. One week later, count the numbers of tumorsphere by using inverted microscope. For tumoursphere-initiating capacity assay, 3,000 cells or 5,000 cells of HUH7 and C3A respectively were seeded in Flat Bottom Ultra-Low Attachment 6 well plate and cultured in DMEM/F12 (1:1) supplemented with B-27 Supplement (Thermo Fisher Scientific, IL, USA), 20 ng/ml of EGF and FGF. One week later, after count the numbers of tumorspheres, the tumorspheres were digested into single cells and further seeded in Flat Bottom Ultra-Low Attachment 6 well plate for a second tumoursphere formation assay, repeat this operation three times and count the numbers of tumorspheres.

FACS

For sorting the EpCAM or CD13 high, medium and low cells of HUH7 and C3A cells: 5×10^6 cells were collected and stained with APC-conjugated EpCAM or PE-conjugated CD13 antibodies (BioLegend, CA, USA). The cells washed two times with PBS containing 0.1% BSA. The cells were resuspended in 1 ml PBS containing 0.1% BSA and sorted by MOFLO XDP system (BECKMAN COULTER, USA).

For analysis the expression of EpCAM or CD13 in HUH7 cells: after treatment, 0.5×10^6 cells of HUH7 were collected and stained with APC-conjugated EpCAM or PE-conjugated CD13 antibodies (BioLegend, CA, USA). The cells washed two times with PBS containing 0.1% BSA. The cells were resuspended in 0.5 ml PBS containing 0.1% BSA and analysed by FC500 MPL system (BECKMAN COULTER, USA).

Supplemental Figures and Figure Legends

Supplemental Figure S1

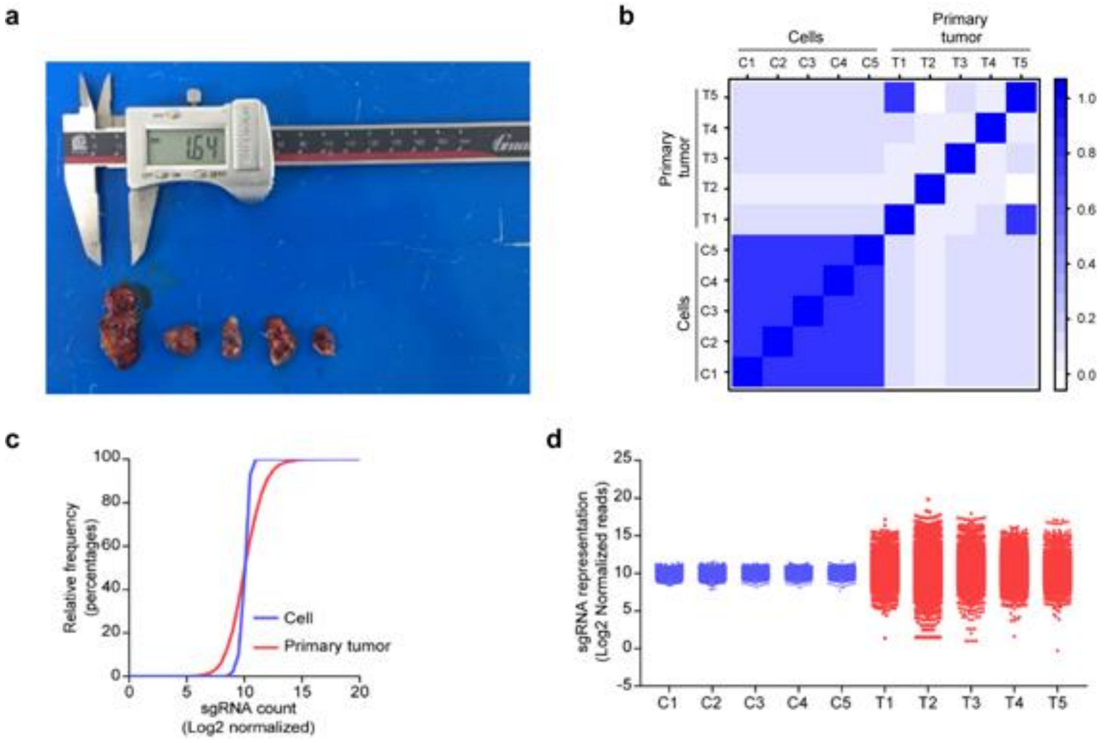

Figure S1. *In vivo* CRISPR/Cas9 screens identify metabolic genes that determine HCC cell tumorigenicity.

(**a**) Xenograft tumors formed subcutaneously in the mice. (**b**) Rank correlation of normalized sgRNA read count from HUH7 cell biological replicates before transplantation and primary xenograft tumors (n= 5). (**c**) Cumulative frequency of library sgRNAs in the cells before transplantation and primary xenograft tumors. A shift in the primary tumor curve represents depletion in a subset of sgRNAs. Distributions for each sample type are averaged across biological replicates. (**d**) Scatterplots showing the distribution of sgRNA frequencies for the cells before transplantation and primary xenograft tumors.

Supplemental Figure S2

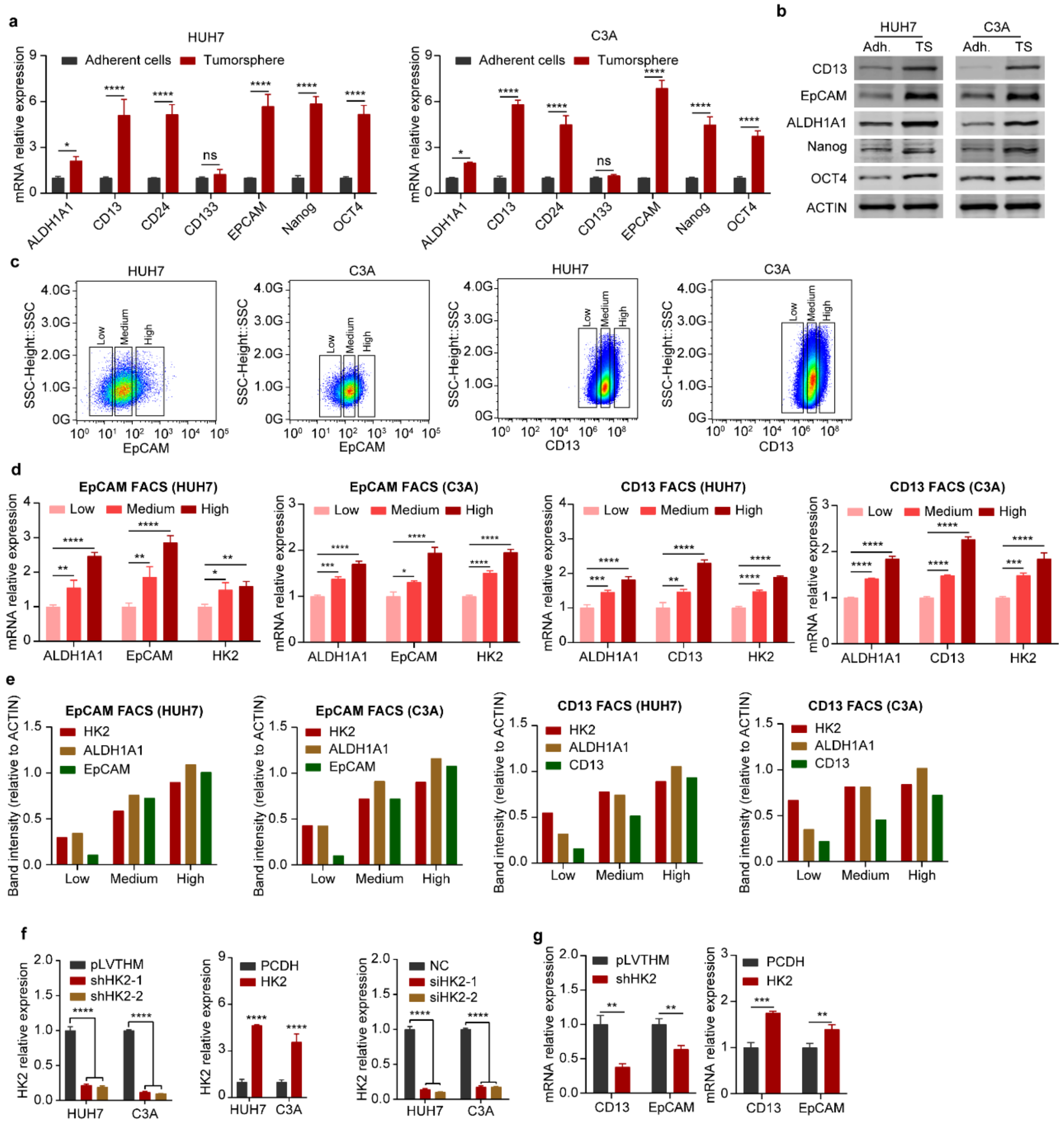

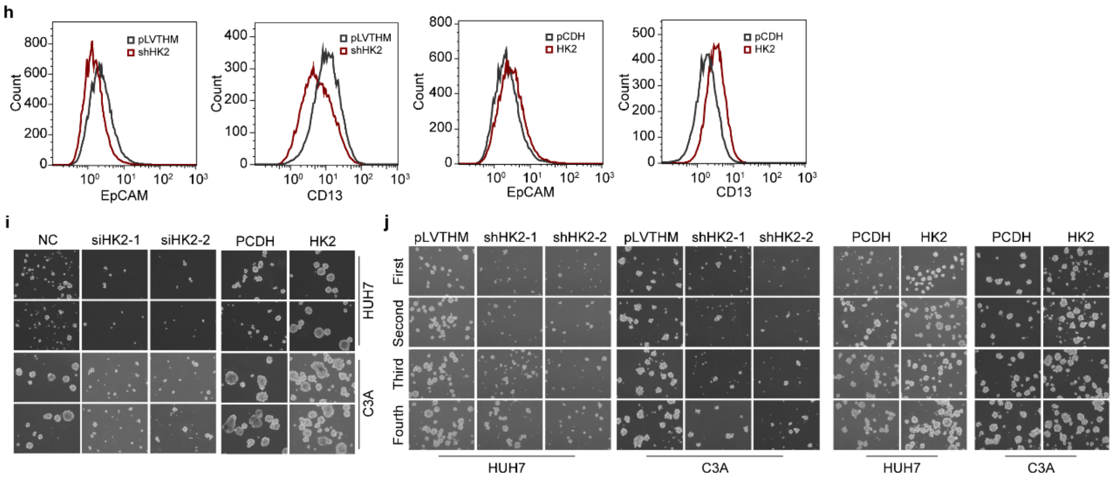

k

	Cell numbers injected	Tumor incidence	Tumor incidence rate (%)
pLVTHM	5x10^6	6/6	100
	1x10^6	5/6	83.3
	0.5x10^6	5/6	83.3
	0.1x10^6	3/6	50
	0.05x10^6	2/6	33.3
shHK2	5x10^6	6/6	100
	1x10^6	5/6	83.3
	0.5x10^6	1/6	16.7
	0.1x10^6	1/6	16.7
	0.05x10^6	0/6	0

l

	Cell numbers injected	Tumor incidence	Tumor incidence rate (%)
pCDH	5x10^6	6/6	100
	1x10^6	5/6	83.3
	0.5x10^6	5/6	83.3
	0.1x10^6	2/6	33.3
	0.05x10^6	1/6	16.7
HK2	5x10^6	6/6	100
	1x10^6	6/6	100
	0.5x10^6	5/6	83.3
	0.1x10^6	4/6	66.7
	0.05x10^6	3/6	50

Figure S2. HK2 is highly expressed in liver CSCs and is essential for maintaining their stemness and self-renewal

(**a**) Real-time qPCR showing the mRNA expression of a series of CSC markers (ALDH1A1, CD13, CD24, CD133, EpCAM, Nanog and OCT4) (relative to ACTB) in adherent cells and the tumorspheres of HUH7 (left) and C3A (right) cells. Data are represented as the mean ± SD (n = 3). Unpaired t-test. (**b**) Western blot measuring the protein levels of a series of CSC markers (ALDH1A1, CD13, EpCAM, Nanog and OCT4) in adherent cells and the tumorspheres of HUH7 and C3A cells. Actin was detected as a loading control. (**c**) FACS to sort HUH7 or C3A cells with APC-EpCAM antibody or PE-CD13 antibody staining. In order to harvest low, medium, and high of CD13 or EpCAM population, we set gate on Low, medium, and high of CD13 or EpCAM population count for the 30% of total cells for separating them as much as possible. (**d**) Real-time qPCR showing ALDH1A1, EpCAM and HK2 mRNA expression (relative to ACTB) in HUH7 and C3A cells after sorting by FACS with

APC-EpCAM antibody or PE-CD13 antibody staining. Data are represented as the mean ± SD (n = 3). One-way ANOVA with multiple comparisons correction. (**e**) The blot quantification (relative to ACTIN) of HK2, ALDH1A1 and EpCAM in EpCAM-expressed low, medium and high groups of HUH7 and C3A cells, which was obtained by Image J and normalized by the internal control (ACTIN) (n = 1). (**f**) Real-time qPCR showing HK2 mRNA expression (relative to ACTB) in HUH7 and C3A cells after HK2 knockdown with shRNAs (left) or siRNAs (right) or HK2 overexpression (middle). Data are represented as the mean ± SD (n = 3). One-way ANOVA with multiple comparisons correction. (**g**) Real-time qPCR showing CD13 and EpCAM mRNA expression (relative to ACTB) in HUH7 and C3A cells after HK2 knockdown or HK2 overexpression, respectively. Data are represented as the mean ± SD (n = 3). Unpaired t-test. (**h**) FACS analysis of the number of EpCAM+ or CD13+ HUH7 cells after HK2 knockdown or HK2 overexpression. (**i**) Tumorsphere formation assay of HUH7 and C3A cells after HK2 knockdown or HK2 overexpression. n = 2. Additionally, see the statistics in Figure 2g and 2h. (**j**) A serial tumorsphere formation assay of HUH7 and C3A cells after HK2 knockdown or HK2 expression. Additionally, see the photographs in Figure 2i and 2j. Data are represented as the mean ± SD (n = 2). One-way ANOVA with multiple comparisons correction. (**k**) Statistical data sheet of Figure 2k. (**l**) Statistical data sheet of Figure 2l. ns, non-significant, *p < 0.05, **p < 0.01, ***p < 0.001, ****p < 0.0001.

Supplemental Figure S3

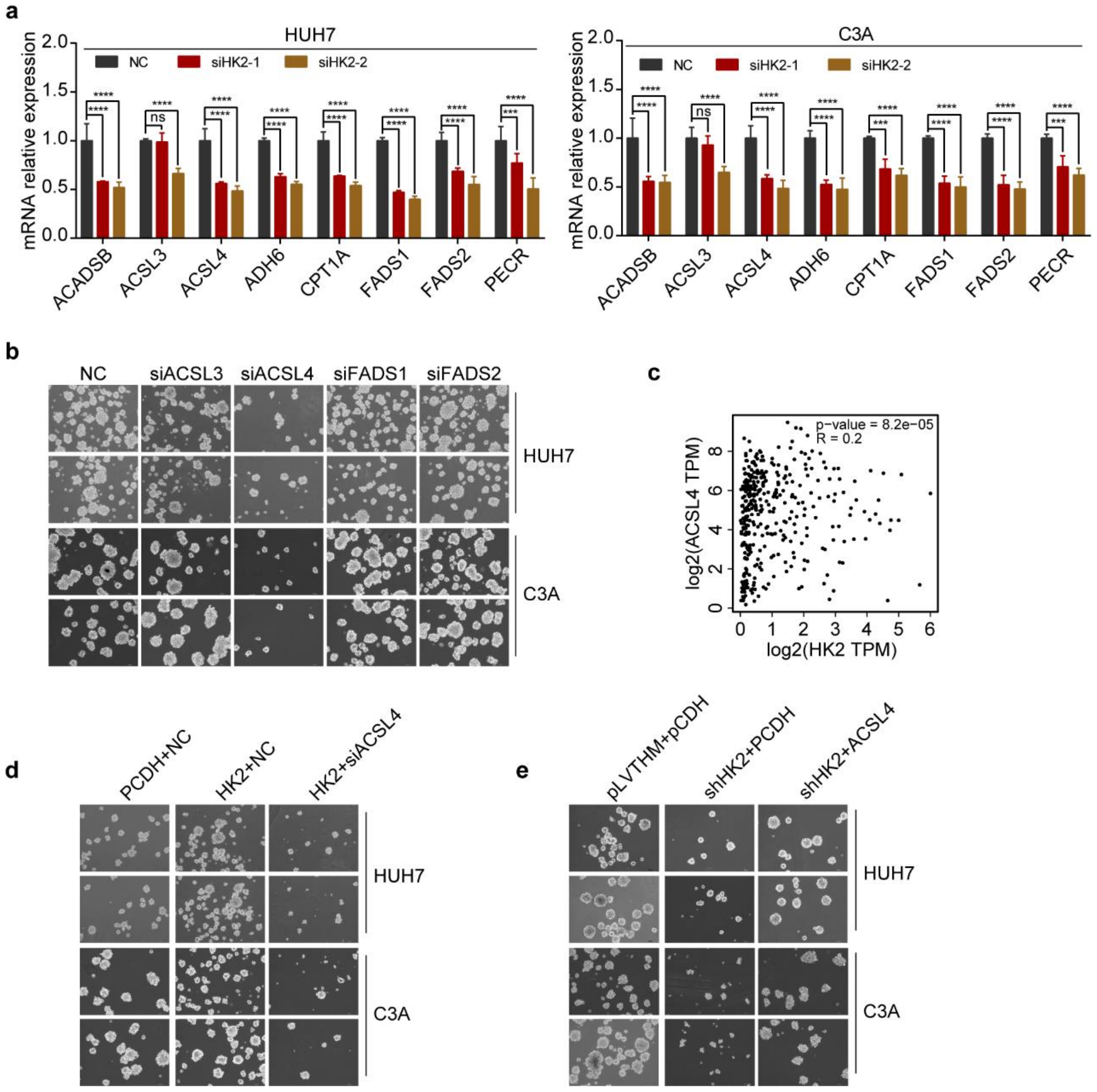

Figure S3. ACSL4 is required for HK2-maintained liver CSC stemness

(**a**) Real-time qPCR showing fatty acid metabolism- and fatty acid degradation-related gene mRNA expression (relative to ACTB) in HUH7 (left) and C3A (left) cells after HK2 knockdown. Data are represented as the mean ± SD (n = 3). One-way ANOVA with multiple comparisons correction. (**b**) Tumorsphere formation assay of HUH7 (up) and C3A (down) cells after transfection with NC, ACSL3, ACSL4, FADS1 and FADS2 siRNAs. Additionally, see the statistics in Figure 3e. (**c**) The correlation

between the expression of HK2 and ACSL4 in human HCC. This analysis used the RNA-seq data of TCGA-LIHC with spearman correlation coefficient on GEPIA online database. (**d**, **e**) Tumorsphere formation assay of HUH7 and C3A cells after HK2 overexpression and ACSL4 knockdown (**d**) or HK2 knockdown and ACSL4 overexpression (**e**). Additionally, see the statistics in Figure 3j. ns, non-significant, *$p < 0.05$, **$p < 0.01$, ***$p < 0.001$, ****$p < 0.0001$.

Supplemental Figure S4

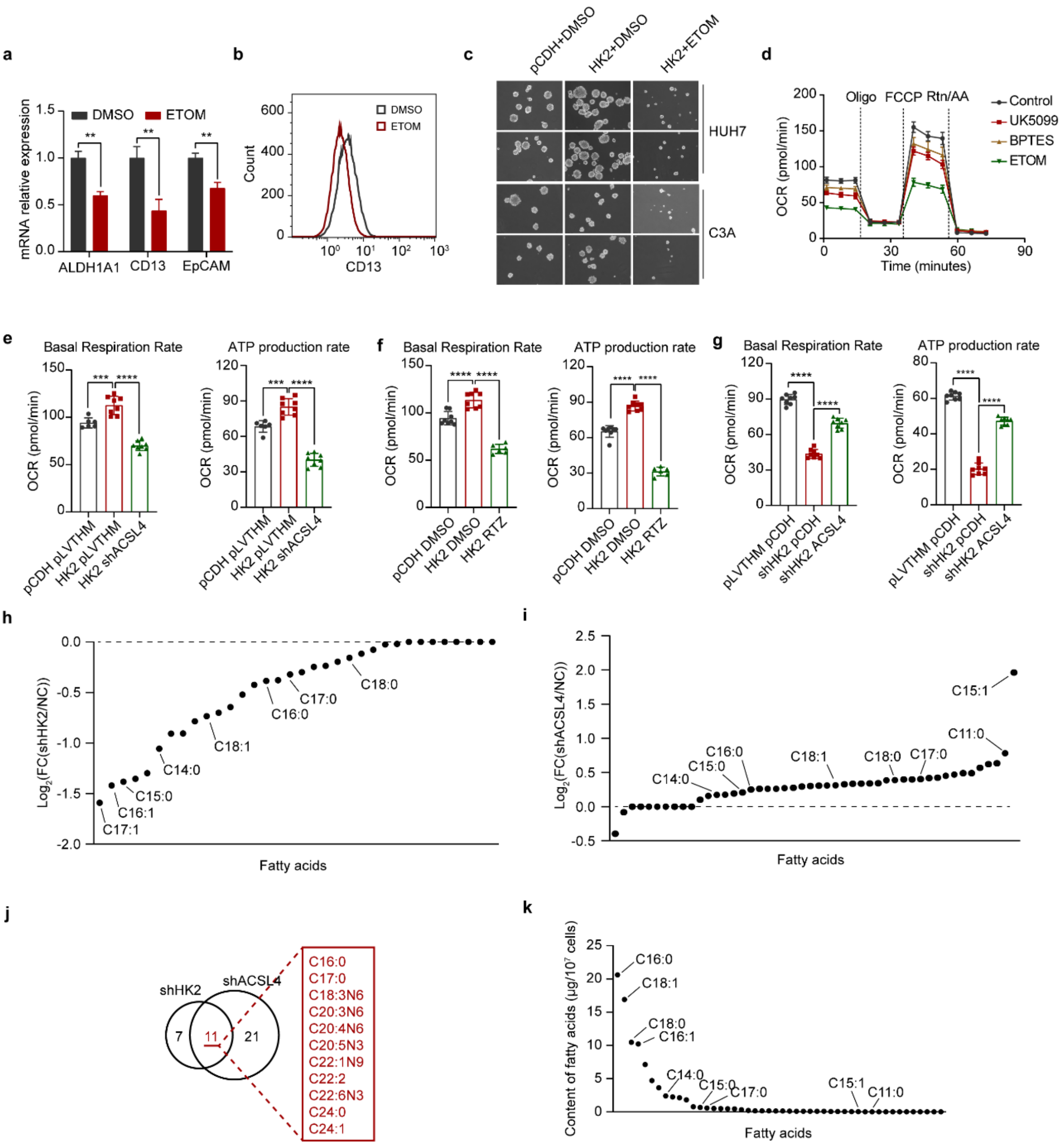

Figure S4. ACSL4-activated FAO-mediated HK2-regulated stemness of liver CSCs

(**a**) Real-time qPCR showing CD13, EpCAM and ALDH1A1 mRNA expression (relative to ACTB) in HUH7 cells after treatment with DMSO or 10 μM ETOM. Data are represented as the mean ± SD (n = 3). One-way ANOVA with multiple comparisons correction. (**b**) FACS was used to analyze the number of CD13+ HUH7 cells after treatment with DMSO or 10 μM ETOM. (**c**) Tumorsphere formation assay

of HUH7 (top) and C3A (down) cells after HK2 overexpression and treatment with ETOM, n = 2 wells per condition. Additionally, see the statistics in Figure 4b. (d) Time series of OCR measurements in BPTES-, UK5099- or ETOM-treated HUH7 cells by a Seahorse Metabolic Analyzer. Data are represented as the mean ± SD, n = 13 wells per condition. (**e**) All groups of Figure 4G basal respiration rates (left) and mitochondrial ATP production rates (right). n = 7–8 wells per condition. One-way ANOVA with multiple comparisons correction. (**f**) All groups of Figure 4H basal respiration rates (left) and mitochondrial ATP production rates (right). n = 7–8 wells per condition. One-way ANOVA with multiple comparisons correction. (**g**) All groups of Figure 4I basal respiration rates (left) and mitochondrial ATP production rates (right). n = 7–8 wells per condition. One-way ANOVA with multiple comparisons correction. (**h**) Mass spectrum (MS) assay to quantify fatty acids in HCC cells after HK2 knockdown. (**i**) Mass spectrum (MS) assay to quantify fatty acids in HCC cells after ACSL4 knockdown. (**j**) Venn diagram showing the decreased fatty acids in shHK2 groups vs NC groups (log2(shHK2/NC) < -0.25) and accumulated fatty acids in shACSL4 groups vs NC groups (log2(shACSL4/NC) > 0.25). The 11 common fatty acids were showed in the red box on the right. (**k**) Analysis of the content of fatty acids in HCC cells. ns, non-significant, *p < 0.05, **p < 0.01, ***p < 0.001, ****p < 0.0001.

Supplemental Figure S5

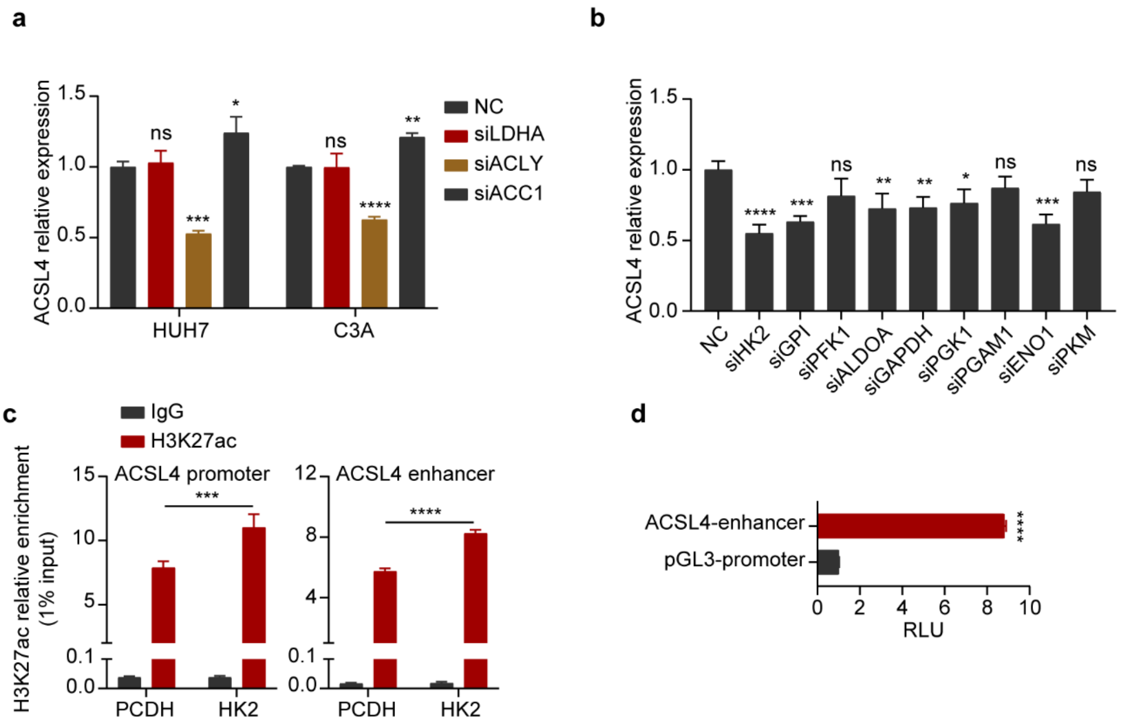

Figure S5. Acetyl-CoA accumulation by HK2 facilitates the enhancer and transcriptional activity of ACSL4

(**a**) Real-time qPCR showing ACSL4 mRNA expression (relative to ACTB) in HUH7 and C3A cells after transfection with NC, LDHA, ACLY or ACC1 siRNAs as indicated above. Data are represented as the mean ± SD (n = 3). One-way ANOVA with multiple comparisons correction. (**b**) Real-time qPCR showing ACSL4 mRNA expression (relative to ACTB) in HUH7 cells after glycolytic enzymes. Data are represented as the mean ± SD (n = 3). One-way ANOVA with multiple comparisons correction. (**c**) ChIP-PCR showing H3K27ac enrichment (relative to 1% input) at the ACSL4 promoter (left) and enhancer (right) regions in HUH7 cells after HK2 overexpression. Data are represented as the mean ± SD (n = 3). Two-way ANOVA with multiple comparisons correction. (**d**) Luciferase assay showing the enhancer

activity of the ACSL4 enhancer region. RLU, relative luciferase unit. Data are represented as the mean ± SD ($n = 3$). Unpaired t-test. ns, non-significant, *$p < 0.05$, **$p < 0.01$, ***$p < 0.001$, ****$p < 0.0001$.

Supplemental Figure S6

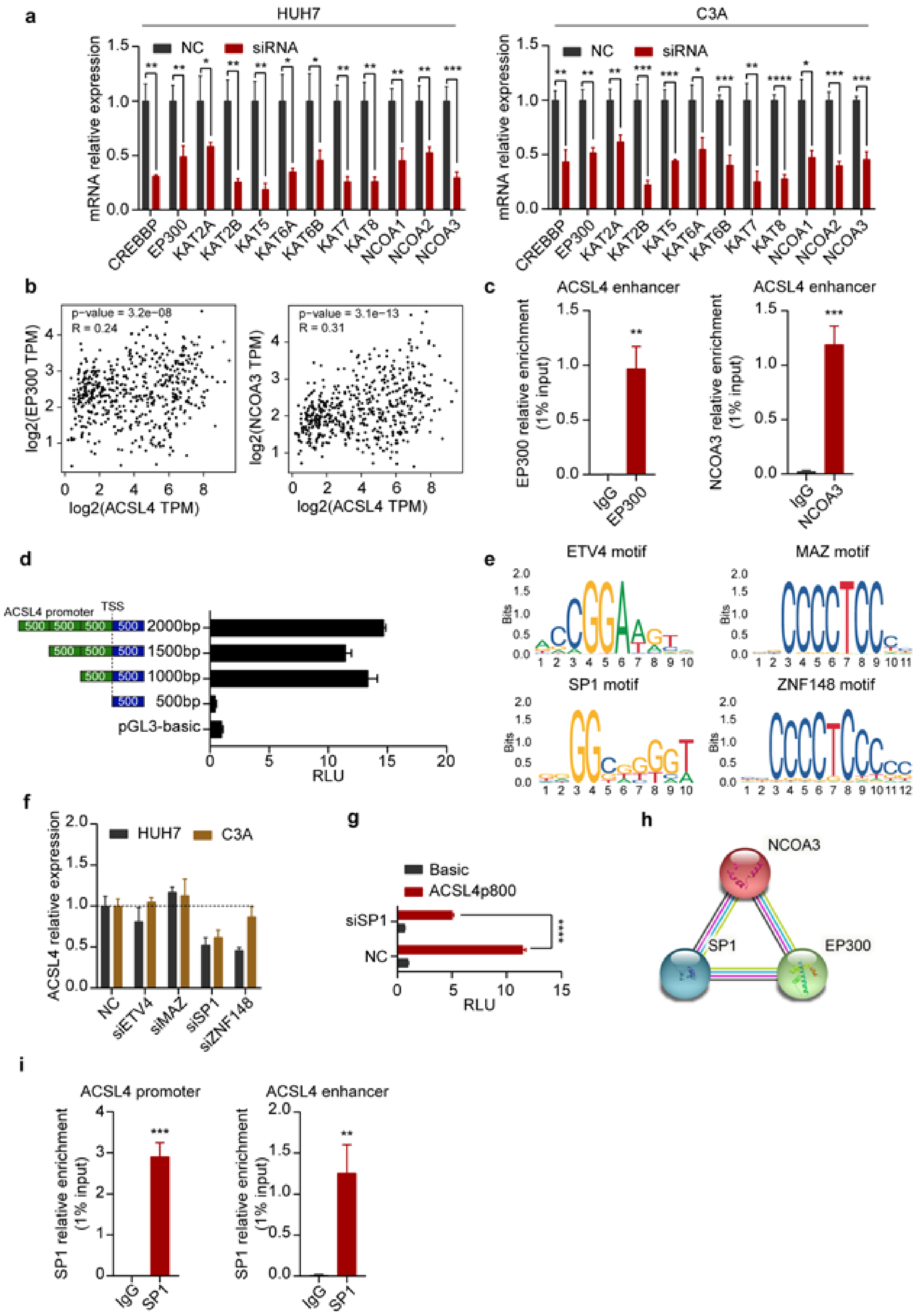

Figure S6. EP300, NCOA3, and SP1 are required for the activation of ACSL4 transcription

(**a**) Real-time qPCR showing the efficiency of a series of acetyltransferase siRNAs in both HUH7 (left) and C3A (right) cells. Data are represented as the mean ± SD (n = 3). Unpaired t-test. (**b**) Scatter plot showing the expression correlation of ACSL4 and EP300 (left) or NCOA3 (right) in the TCGA-LIHC cohort in the GEPIA database. (**c**) ChIP-PCR showing EP300 (left) and NCOA3 (right) enrichment (relative to 1% input) at ACSL4 enhancer regions in HUH7 cells. Data are represented as the mean ± SD (n = 3). Unpaired t-test. (**d**) Luciferase assay measuring the active regions of the ACSL4 promoter in HUH7 cells by using a series of truncated regions of the ACSL4 promoter. Schematic diagram on the left. TSS, transcription start site. RLU, relative luciferase unit. Data are represented as the mean ± SD (n = 3). (**e**) Schematic diagram showing the binding motif of 4 candidate transcription factors. (**f**) Real-time qPCR showing ACSL4 mRNA expression (relative to ACTB) in HUH7 and C3A cells after transfection with ETV4, MAZ, SP1 and ZNF148 siRNAs. Data are represented as the mean ± SD (n = 3). One-way ANOVA with multiple comparisons correction. (**g**) Luciferase assay measuring ACSL4 promoter activity in HUH7 cells after SP1 knockdown. RLU, relative luciferase unit. Data are represented as the mean ± SD (n = 3). Two-way ANOVA with multiple comparisons correction. (**h**) STRING analysis showing the interactions of EP300, NCOA3 and SP1. (**i**) ChIP-PCR showing SP1 enrichment (relative to 1% input) at the ACSL4 promoter (left) and enhancer (right) regions in HUH7 cells. Data are represented as the mean ± SD (n = 3). Unpaired t-test. ns, non-significant, *$p < 0.05$, **$p < 0.01$, ***$p < 0.001$, ****$p < 0.0001$.

Supplemental Figure S7

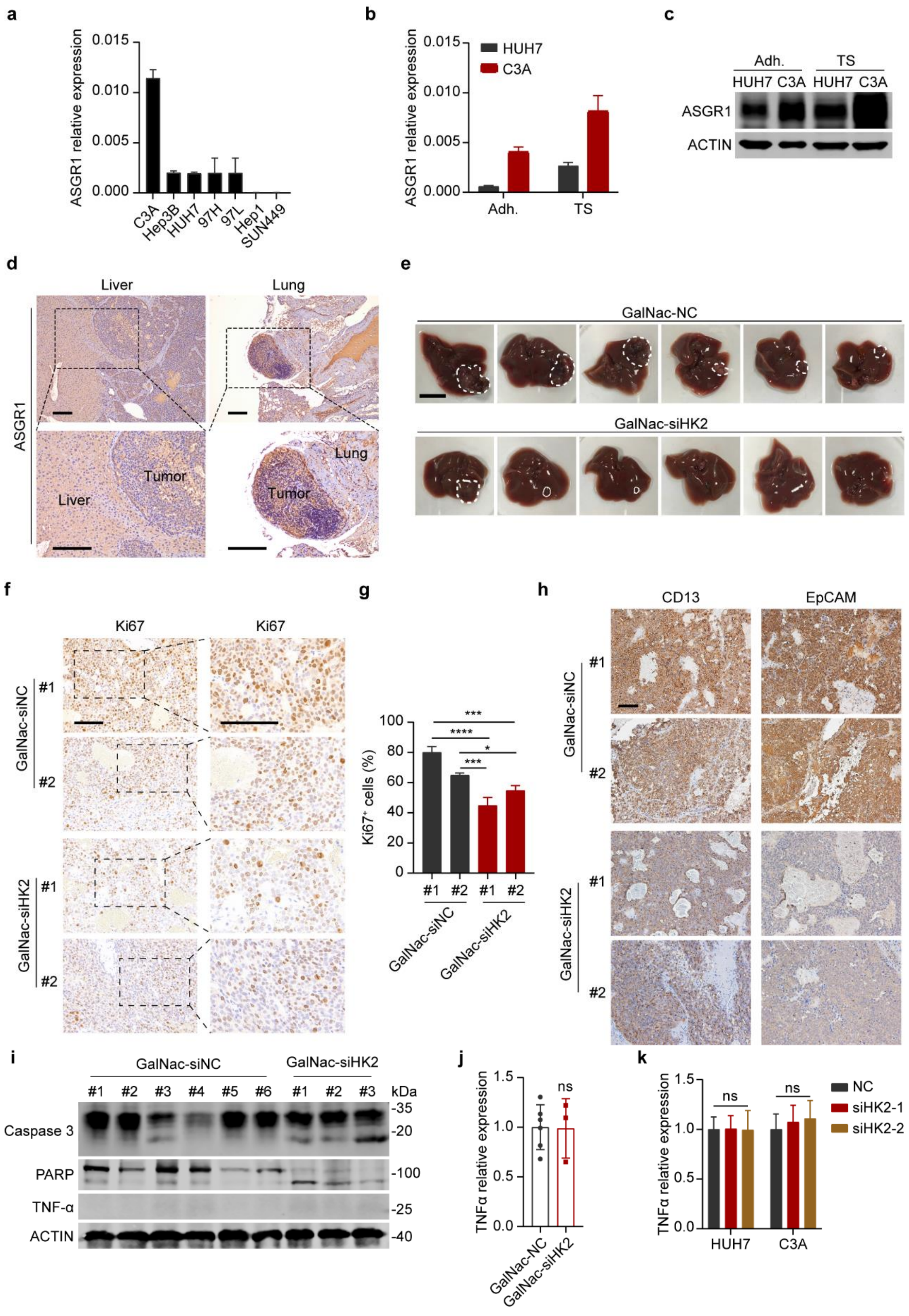

Figure S7. ASGR1 is expressed in a mouse liver cancer model.

(**a**) Real-time qPCR showing ASGR1 mRNA expression (relative to ACTB) in a series of liver cancer cell lines. Data are represented as the mean ± SD (n = 3). (**b**) Real-time qPCR showing ASGR1 mRNA expression (relative to ACTB) in adherent cells (adh.) and tumorspheres of HUH7 and C3A cells. Data are represented as the mean ± SD (n = 3). (**c**) Western blots measuring ASGR1 expression in adherent cells (adh.) and tumorspheres of HUH7 and C3A cells. (**d**) IHC of ASGR1 in HUH7-formed liver cancer samples and lung tissue with liver cancer metastasis. All scale bars represent 100 μm. (**e**) Pictures of the liver showing the tumors of the negative control and GalNac-siHK2 treatment groups (n=6). Tumors in the liver are highlighted with white lines. Scale bars represent 1 cm. (**f**) Images of Ki67 staining. Scale bar, 100 μm. (**g**) Statistic analysis of $Ki67^+$ cells in Ki67 staining images. (**h**) IHC staining of CSC markers, CD13 and EpCAM, in negative control and siHK2 knockdown mouse tumor samples. Scale bar, 100 μm. (**i**) Western blots measuring Caspase-3, PARP and TNFα expression in the tumors from GalNac-siNC group and GalNac-siHK2 group. ACTIN was detected as a loading control. (**j**) Real-time qPCR showing TNFα mRNA expression (relative to ACTB) in the tumors from GalNac-siNC group and GalNac-siHK2 group. Data are represented as the mean ± SD (n = 3~6). Unpaired t-test. (**k**) Real-time qPCR showing TNFα mRNA expression (relative to ACTB) in HUH7 and C3A cells after HK2 knockdown. Data are represented as the mean ± SD (n = 3). One-way ANOVA with multiple comparisons correction. ns, non-significant.

Supplemental Figure S8

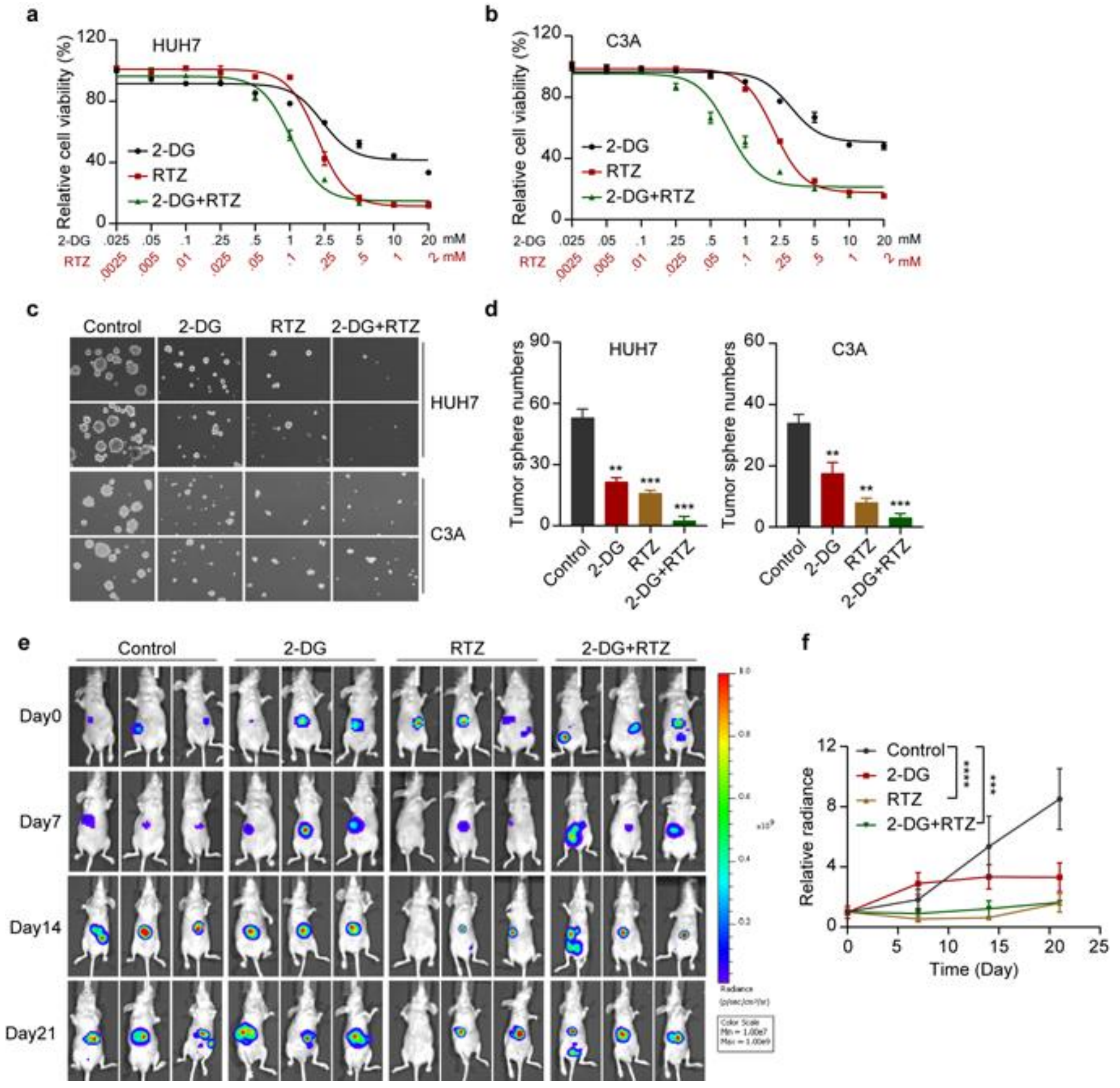

Figure S8. An inhibitor of HK2 or ACSL4 dampens liver cancer cell growth in vitro and *in vivo*.

(**a**, **b**) CCK-8 assay to analyze the viability of HUH7 (A) and C3A (B) cells treated with 2-DG, RTZ or 2-DG + RTZ. The treatment concentrations of 2-DG and RTZ are indicated as black and red words. Data are represented as the mean ± SD (n = 2). (**c**, **d**) Tumorsphere formation assay of HUH7 and C3A cells treated with 2-DG, RTZ or 2-DG + RTZ. Images are shown in (c), and statistics are shown in (d). Data are

represented as the mean ± SD (n = 2). One-way ANOVA with multiple comparisons correction. (**e**) HUH7 cells stably expressing GFP-luciferase were injected into the liver in situ of BALB/c nude mice. Seven days later, the mice were separated into four groups (n=6) and gavaged with 2-DG (100 mg/kg), RTZ (5 mg/kg) or 2-DG + RTZ every day. *In vivo* living imaging to analyze the tumor growth. (**f**) Statistics of the relative radiance abundance of *in vivo* living images. Data are represented as the mean ± SD (n = 3). Two-way ANOVA with multiple comparisons correction. ns, non-significant, *p < 0.05, **p < 0.01, ***p < 0.001, ****p < 0.0001.
